# Drivers of arthropod biodiversity in an urban ecosystem

**DOI:** 10.1038/s41598-023-50675-3

**Published:** 2024-01-03

**Authors:** Jayme M. M. Lewthwaite, Teagan M. Baiotto, Brian V. Brown, Yan Yin Cheung, Austin J. Baker, Charles Lehnen, Terrence P. McGlynn, Vaughn Shirey, Lisa Gonzalez, Emily Hartop, Peter H. Kerr, Eric Wood, Laura Melissa Guzman

**Affiliations:** 1 Marine and Environmental Section, Department of Biological Sciences, University of Southern California, Los Angeles, 90089 USA; 2 Department of Entomology, Natural History Museum of Los Angeles County, Los Angeles, 90007 USA; 3 Human Evolutionary Biology Section, Department of Biological Sciences, University of Southern California, Los Angeles, 90089 USA; 4 Department of Biology, California State University Dominguez Hills, Carson, 90747 USA; 5 Department of Biology, Georgetown University, Washington, DC 20057 USA; 6 Natural History Museum of Los Angeles County, Los Angeles, 90007 USA; 7 Center for Integrative Biodiversity Discovery, Museum für Naturkunde, Berlin, Germany; 8 California State Collection of Arthropods, CDFA Plant Pest Diagnostics Center, Sacramento, CA 95832 USA; 9 Department of Biological Sciences, California State University Los Angeles, 5151 State University Drive, Los Angeles, 90032 USA

**Keywords:** Urban ecology, Ecological modelling

## Abstract

Our world is becoming increasingly urbanized with a growing human population concentrated around cities. The expansion of urban areas has important consequences for biodiversity, yet the abiotic drivers of biodiversity in urban ecosystems have not been well characterized for the most diverse group of animals on the planet, arthropods. Given their great diversity, comparatively small home ranges, and ability to disperse, arthropods make an excellent model for studying which factors can most accurately predict urban biodiversity. We assessed the effects of (i) topography (distance to natural areas and to ocean) (ii) abiotic factors (mean annual temperature and diurnal range), and (iii) anthropogenic drivers (land value and amount of impervious surface) on the occurrence of six arthropod groups represented in Malaise trap collections run by the BioSCAN project across the Greater Los Angeles Area. We found striking heterogeneity in responses to all factors both within and between taxonomic groups. Diurnal temperature range had a consistently negative effect on occupancy but this effect was only significant in Phoridae. Anthropogenic drivers had mixed though mostly insignificant effects, as some groups and species were most diverse in highly urbanized areas, while other groups showed suppressed diversity. Only Phoridae was significantly affected by land value, where most species were more likely to occur in areas with lower land value. Los Angeles can support high regional arthropod diversity, but spatial community composition is highly dependent on the taxonomic group.

## Introduction

Urban ecosystems, including the developed infrastructure of towns and cities, are among the most widespread forms of land use globally^1^. Cities are novel, highly manicured ecosystems composed of developed structures intermixed with vegetation and impervious surfaces where human populations are the main drivers of change^2,3^. Thus, the traditional view of urban ecosystems is that they are generally devoid of biodiversity, especially when compared with natural areas. Nevertheless, a surge of research over the past few decades has highlighted the critical role of cities in harboring distinct flora and fauna—especially for vertebrates such as birds and mammals, which have been the predominant focus of urban wildlife study^4–8^. Invertebrates, including arthropods, which are the most diverse group of animals on the planet, have been comparably less studied^9^. For example, despite recent efforts in describing new insect species within urban ecosystems, the number of insects in urban environments, the extent of their ranges, and other aspects of their life history are generally unknown^10^. One exception to this is urban pollinator research, which has rapidly grown in scope and volume in recent years. This research has shown that urban areas can act as refuges^11^ for pollinators when there is adequate suitable habitat^12^, but that these habitats disproportionately favor generalist species^13^. Given the ecological importance of arthropods in virtually all terrestrial ecosystems, and considering the widespread declines in arthropods across the globe, it is imperative to expand the taxonomic scope of urban ecology research to describe the distribution and ecology within cities in lesser-studied arthropod groups.

Characterizing the drivers of arthropod distributions in cities is essential because of their critical role in urban ecosystems. They are integral components of food webs that provide a broad range of ecosystem services in cities^14^. When food webs in cities become simplified, the functional roles of the key players are amplified^15^. Considering that arthropods fill many trophic roles, maintaining arthropod biodiversity is imperative for many other organisms. For example, they are a critically important food source for urban bird species. Cities can serve as reservoirs of pollinator diversity, and green areas within them can serve as climatic “stepping stones” to connect habitats across the landscape^16–18^. Additionally, arthropods are important for decomposition processes, nutrient cycling, and biological control of pests^19^. They also provide a window into other taxa that receive even less attention in urban ecology, such as saprotrophic organisms^20^ and cryptic soil biota^21^. While there are many immediate steps that we can take to protect and foster urban arthropod biodiversity^22^, more effective policies and practices require a more nuanced understanding of the characteristics of urban environments that support a diverse and resilient arthropod fauna^23^, as well as the taxonomic groups most susceptible to increasing urbanization^24^.

Arthropod distributions in urban environments can be driven by a wide variety of factors, including climate-related variables, as well as income and human-related variables. Disentangling the effects of each of these becomes challenging, particularly in urban areas where micro-climates and micro-habitats depend on both large-scale climate patterns, as well as anthropogenic structures. For example, the amount of water available for arthropods depends both on the amount of precipitation, as well as the irrigation patterns in a given area. Using only observational data, ecologists have often relied on techniques like model selection in hopes of extracting cause-and-effect relationships. However, these techniques can lead to inaccurate or even biased results^25,26^. For example, the inclusion of a third variable (Z) in a model can unintentionally remove the causal effect between X and Y if there is a causal link between X and Z that is unaccounted for^27^. Causal inference approaches have emerged as a powerful tool to overcome this hurdle as they are concerned with predicting the consequences of intervening in a system (e.g., how X impacts Y) by considering the effect additional variables (e.g. Z) could have on causal relationships between X and Y^28^. Essentially, the framework involves (i) identifying variables that are likely important in the system; (ii) making causal assumptions about their relationships between variables, (iii) identifying which effects to estimate; and (iv) exploring the consequences of changing the assumptions to see whether inferences hold under different sets of assumptions^25^.

The greater Los Angeles metropolitan area (L.A.) is one of the largest urbanized areas in the United States. Due to the development patterns, in many places throughout the city, it is a short distance between the surrounding mountainous natural areas and high-density urban areas (e.g. Santa Monica Mountains to downtown and Century City), which creates a narrow urban-to-natural gradient. Along this gradient, numerous factors shift, including elevation, mean and diurnal temperature, and income distribution (via the luxury hypothesis described below), that likely affect arthropod species distributions. Although these narrow gradients may suppress diversity in many taxonomic groups, they could alternatively create high regional diversity (along with high beta diversity) and abundance levels in arthropods due to their relatively small home range sizes, high sensitivity to climatic drivers, and the variety of micro-habitats found in urbanized landscapes. For example, temperature has been shown to explain spatial insect beta diversity gradients^29^. Furthermore, urban structures can either increase^30^ or decrease^24^ insect richness, depending on the interplay between densification vs. sprawl as well as the specific life history requirements of the group^31,32^.

Beyond climatic drivers, local-scale influences like urban green areas can dramatically affect arthropod occurrence^33^. However, L.A. has a long and fraught history of real estate inequity, which has left a legacy on land value across the city^34^ where high-income neighborhoods have disproportionately higher levels of vegetation cover and green area than lower-income neighborhoods^35^. This phenomenon is known as the “luxury effect” and hypothesizes a positive relationship between wealth and biodiversity within urban areas and has been demonstrated in groups such as birds^36^, bats^37^, and terrestrial mammals^38^. However, although it may directly impact the amount of habitat and thus the likelihood of occurrence of many arthropod species, very little research to date has examined the luxury effect on arthropod biodiversity^39^.

In this study, we characterize arthropod diversity within 6 different groups across an extensive metropolitan area that spans climatic and urban gradients. Specifically, we sought to disentangle the relative importance of (i) topography (distance to natural areas and distance to ocean) (ii) abiotic factors (mean annual temperature and diurnal range, and relative humidity), and (iii) anthropogenic drivers (land value and amount of impervious surface as proxies for luxury effect) in determining the distributions of individual species as well as comparing responses within and among several major taxonomic groups. We accomplished this with the development of a large-scale and extensive monitoring network across L.A., which sampled arthropods from 2014 to 2018 in various phases, and the application of multi-species occupancy modeling and causal inference tools.

## Results

Overall, we found that arthropod diversity patterns varied dramatically across space in L.A. and diverged significantly across the 6 taxonomic groups we examined. Much of this diversity and divergence between groups can be attributed to the geographical variation in topology, environment, and anthropogenic features across this relatively large area and each group’s and species’ individual response to those predictors. This is apparent from the extensive between- and within-group variation that we observed in both the direction and magnitude of predictor importance on arthropod occupancy. Our predictors were all transformed to the same scale in order to enable meaningful comparisons between groups and drivers. Additionally, models were constructed such that they estimate the marginal contribution of each predictor; i.e. predictors are directly attributable and not confounded with other predictors (see “Methods” section).

**Figure 1:**
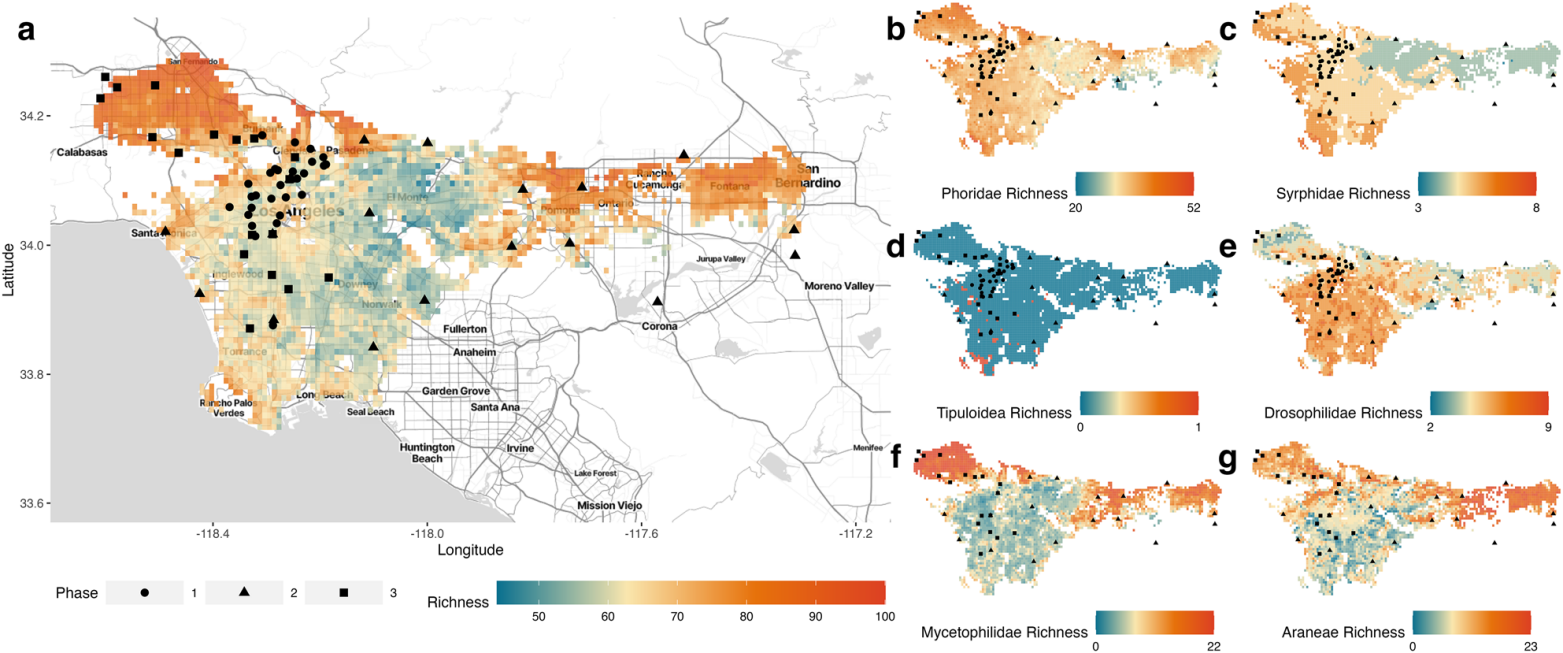
Spatial interpolation of diversity across the greater Los Angeles area. (**a**) Projected richness for all combined groups (n = 236 species) is shown using the continuous color map. (**b–g**) Projected richness is also shown for each individual taxonomic group modelled: Phoridae (n = 108 species), Syrphidae (n = 34 species), Tipuloidea (n = 20 species), Drosophilidae (n = 27 species), Myceotphilidae (n = 23 species), and Araneae (n = 24 species). BioSCAN sampling sites are overlaid with shapes displaying the collection phase (year of sampling). Across groups, total richness is not obviously driven by any single predictor but rather a result of complex group- and species-specific effects. The extent of the spatial map is based on (1) available land value data, which is confined to the Los Angeles and San Bernardino Counties and (2) the bounding of every predictor to 3 standard deviations away from the mean value present at BioSCAN sampling sites. Grid cells are at a 1 km resolution. R packages ggplot2, ggmap, and basemaps were used to create this map. Map tiles by Stamen Design, under CC BY 4.0. Data by OpenStreetMap, under ODbL.

The 60 sites included in this study were widely distributed across L.A.: as far west as the coastline, eastwards to San Bernardino in the San Gabriel Valley, northwards to the San Fernando Valley, and southwards to Long Beach (Fig. 1). The furthest sites were 122 km apart from one another. A large proportion of sites were concentrated in central L.A. In total, 236 species of arthropods were included in our study.

**Figure 2:**
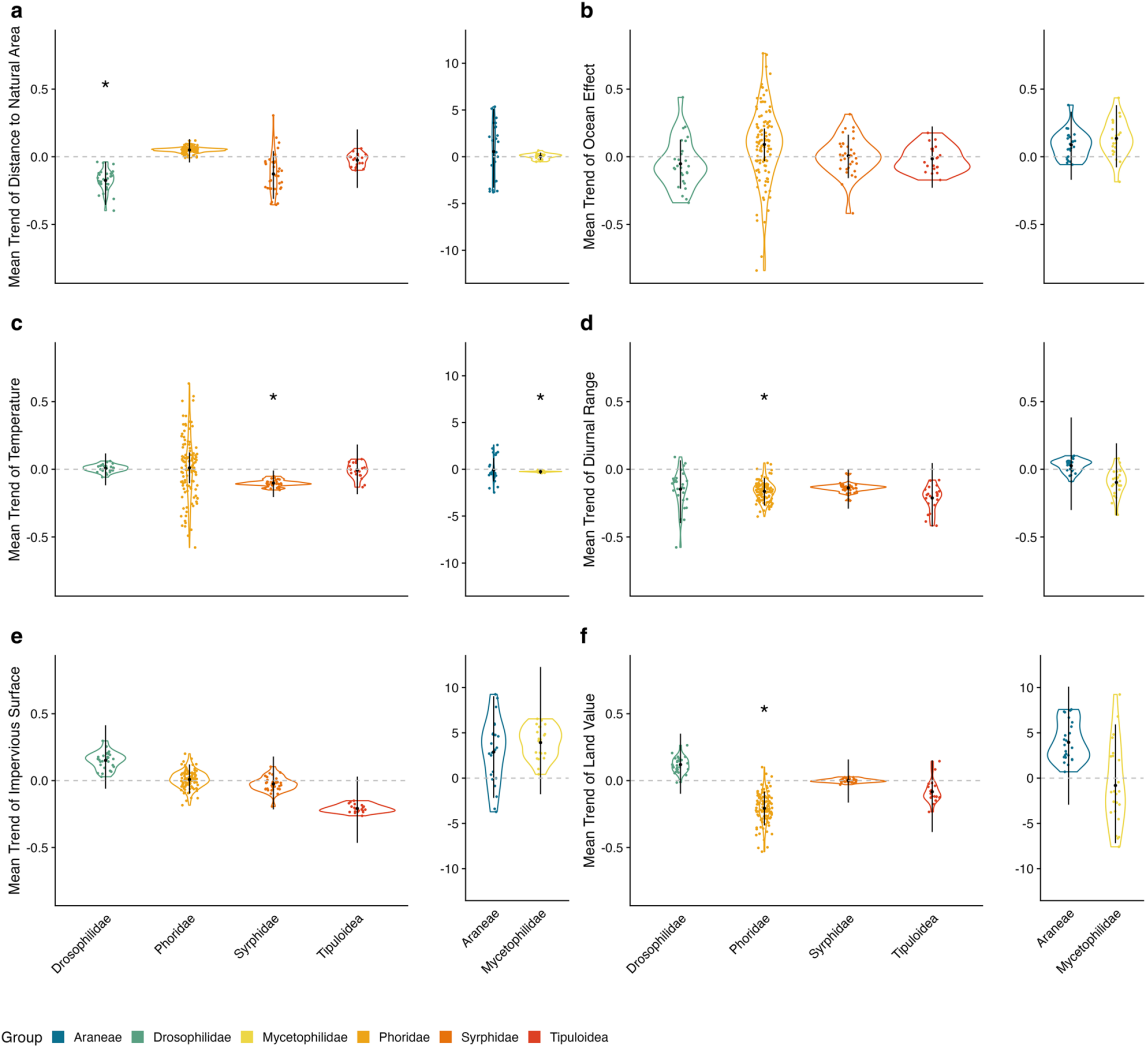
Across-group predictors of occupancy across environmental variables. (**a**) Distance to Natural Area. (**b**) Distance to Ocean. (**c**) Temperature. (**d**) Diurnal Range. (**e**) Impervious Surface. (**f**) Land Value. The six colors displayed in the plots represent different arthropod groups: Araneae (dark blue), Drosophilidae (dark turquoise), Mycetophilidae (yellow), Phoridae (yellow–orange), Syrphidae (orange), and Tipuloidea (red). There are 24, 27, 23, 108, 34, and 20 species in Araneae, Drosophilidae, Mycetophilidae, Phoridae, Syrphidae, Tipuloidea, respectively. Each colored point represent a species from an arthropod group with the same color. The grey horizontal line at y = 0 represents no significant trend. The black point represents the average trend of an arthropod group. The black vertical line represents the 95% confidence interval of the average group trend. Stars above each group represent group-level 95% confidence intervals that do not overlap with zero. Note that the effects of land value and impervious surface are measured within a 0.25 km radius for Phoridae and Araneae, and 0.5 km for Drosophilidae, Syrphidae, Tipuloida and Mycetophilidae. Araneae and Mycetophilidae have different y-axis values for some predictors.

When comparing predictors of occurrence across taxonomic groups, topographical elements consistently had the strongest effects on arthropod occurrence (Fig. 2a,b). Although the mean effect of distance to ocean was centered on 0 and was not statistically significant for all groups, it had the largest within-group variation. Phoridae in particular had a wide variation in responses, with a mean positive effect that overlapped with 0 (0.091, 95% CI [− 0.033, 0.209]) (i.e. many species were more likely to occur farther from the ocean, but this was not universal amongst phorid species). Similarly, distance to natural area had large within- and across-group variation, although this effect was only statistically significant in Drosophilidae (mean estimate of − 0.175, 95% CI [− 0.356, − 0.037]), which were more likely to occur in close proximity to natural areas. Syrphidae occupancy also increased near natural areas, but this was not a statistically significant effect (mean estimate of − 0.127, 95% CI [− 0.311, 0.042]). Meanwhile, there was no significant mean effect on Tipuloidea, Araneae, and Mycetophilidae (Fig. 2a,b), and interestingly, we found a positive (but not significant) effect of distance to natural areas (mean estimate of 0.050, 95% CI [− 0.042, 0.130]) on Phoridae occurrence (i.e. some species were more likely to occur farther from natural areas).

In terms of environmental predictors, the effect of mean annual temperature on species occurrence was not consistent within- or across groups on average, except we found a significant negative relationship in Syrphidae and Mycetophilidae (Fig. 2c,d), indicating that all syrphid and mycetophilid species collected in this study are more likely to occur in cooler sites. Notably, there was a large within-group variation in Phoridae. Meanwhile, mean diurnal temperature range had a consistent and negative effect across all groups, though this effect was only statistically significant in Phoridae. Phorid species were less likely to occur in sites that experienced more extreme fluctuations in temperatures. These results indicate that in L.A., mean temperature on its own does not have a directional effect on species’ distributions, but the diurnal range does.

For anthropogenic predictors of arthropod distributions, there was no mean effect of the amount of impervious surface surrounding a site on occurrence in some groups (Drosophilidae and Phoridae) but a negative effect in other groups (Syrphidae and Tipuloidea) and a positive effect in others (Araneae and Mycetophilidae), though none of these effects were statistically significant (Fig. 2e,f). Similarly, land value had little predictive power in most groups, though interestingly, most Phoridae species were significantly more likely to occur in areas with lower land value (mean estimate of − 0.207, 95 % CI [− 0.334, − 0.083]).

Some of the predictors do not seem to have an overall positive or negative effect on occurrence, but they do correspond to changes in community composition. Although Fig. 1a shows that overall arthropod diversity is lowest in the most urbanized areas of L.A., when this map is subdivided by taxonomic group (Fig. 1b–g), this is not the case for all groups. For example, drosophilid—and to some extent phorid and syrphid diversity—is often higher in central L.A. compared to the outskirts.

**Figure 3:**
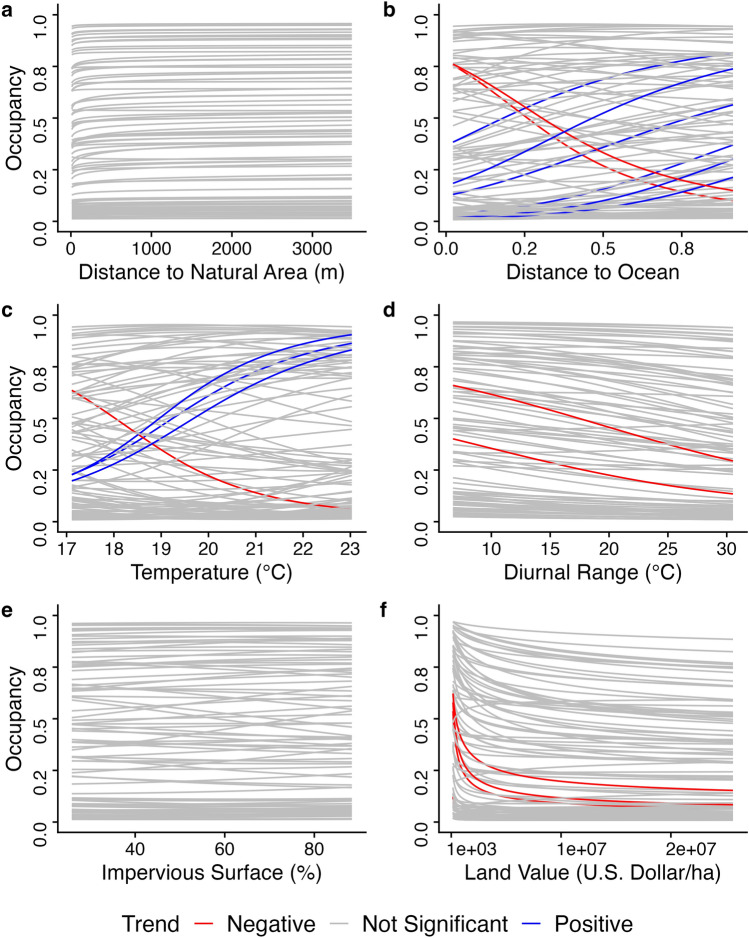
Within-group predictors of occupancy in Phoridae. 108 species are represented in this group. Each plot is showing the single effect of an environmental variable by keeping the other environmental variables in the same model at their average value from the sampling data. (**a**) Distance to Natural Area. (**b**) Distance to Ocean. (**c**) Temperature. (**d**) Diurnal Range. (**e**) Percentage of Impervious Surface. (**f**) Land Value. Each line represents the effect of the environmental variable on an individual species. Red lines indicate a statistically significant negative relationship between the occupancy of a species and an environmental variable, blue lines are statistically significant positive relationships, and grey lines indicate non-significant relationships.

Group-level trends are not representative of all species contained within them, and accordingly, we also characterized within-group variation in responses. We focus on Phoridae in the main text, as they are the most diverse and best-characterized group in this dataset, but similar plots for all other taxonomic groups are located in the Supplemental Material (Supp. Figs. S7–S11). In terms of topographical variables, we found that distance to natural areas had little effect on occupancy (Fig. 3a), while individual phorid species do respond strongly to increasing distance to the ocean. Specifically, two species (*Megaselia donahuei* and *Puliciphora occidentalis*) are significantly more likely to occur at sites near the ocean, while six species (*Diplonevra setigera, Megaselia carthayensis, Megaselia losangelensis, Megaselia simunorum, Megaselia spiniclasper*, and *Trophodeinus furcatus*) are more likely to occur at sites farther from the ocean (Fig. 3b). In terms of climatic variables, we found that both temperature and diurnal range had strong effects on individual species (Fig. 3c,d), but relative humidity did not (Supp. Fig. S6). For temperature, we found three species where occupancy increased significantly with increasing temperature (*Megaselia donahuei, Puliciphora occidentalis*, and *Spiniphora bergenstammii*), and one species where occupancy decreased significantly with increasing temperature (*Megaselia rodriguezorum*, Fig. 3c). The mean effect of diurnal range was negative on Phoridae as a whole, and indeed we found most species to decrease in occupancy as the diurnal range increased, though only two of those species did so significantly (*Megaselia renwickorum* and *Spiniphora bergenstammii* Fig. 3d). Finally, in terms of anthropogenic drivers, we found that impervious surface had a very small and statistically insignificant effect on all individual phorid species (Fig. 3e), but land value had a consistently negative effect. However, only four species decreased significantly as land value increased (*Anevrina variabilis, Megaselia hoggorum, Megaselia scalaris*, and *Phalacrotophora halictorum* Fig. 3f). Further, the decrease of occupancy with increasing land value was most dramatic at the lowest end of the distribution, indicating that above a certain threshold land value, phorid occurrence was no longer impacted. Some of the species listed above show similar responses to seemingly opposing predictors (e.g. *Megaselia donahuei* is more likely to occur at sites near the ocean, and also in sites with higher mean temperatures; whereas mean temperature and distance to ocean negatively covary in space Fig. S15 and S17). We note that these are effects marginal effects, after accounting for possible confounders.

## Discussion

As cities across the world continue to grow at unprecedented rates, determining features of urban ecosystems that support or hinder biodiversity is a critical and pressing need. Here, we used a robust arthropod dataset, coupled with topographic, abiotic, and anthropogenic predictor variables, to uncover drivers of arthropod diversity and occupancy patterns across L.A. Our results highlight the differential responses of arthropods to various geographic and biological features of L.A. L.A. is surrounded by the Pacific Ocean—to the west and south—and mountains—to the north and east—that are primarily protected lands. Therefore, arthropods may be more diverse the closer they are to potential source populations in the protected areas. Abiotic factors were also key drivers of arthropod diversity patterns where the mean diurnal temperature range at a given location was a stronger predictor than average temperature values. Given that arthropods are ectothermic, species range limits may be governed by their physiological tolerances for a range of temperature conditions. Lastly, anthropogenic variables, such as land cover, were less strong in their predictive power, which is in contrast to studies of birds and other taxa^36^, bats^37^, and terrestrial mammals^38^. Taken together, our study uncovers important relationships among predictor and response variables and highlights potential important paths for future research.

One of our most important findings was the large heterogeneity in responses both within taxonomic groups as well as across them. For example, land value and amount of impervious surface had opposing trends in Drosophilidae compared to Tipuloidea. Meanwhile, at the individual species level, there is large variation within phorids in their probability of occurring in close proximity to the ocean. However, some trends were broadly consistent: larger diurnal temperature ranges negatively impacted occurrence in almost all species (Fig. 2d), indicating this is a strong limiting factor in L.A.’s arthropod biodiversity.

All of the heterogeneity in species-level responses scales up to create dramatic spatial variation in arthropod biodiversity in L.A. (Fig. 1). Although some groups have high levels of diversity in the densely urbanized areas of central L.A. (e.g. Drosophilidae), other groups are most diverse in the city’s outskirts. In some groups such as spiders, these patterns may be driven by invasive species (a number of which are present in our dataset), who tend to do better in low-diversity systems^40^. We note that predicted species richness for Tipuloidea and Syrphidae maps is consistently low (even though our study includes 34 Syrphidae species and 20 Tipuloidea species). This could potentially be driven by low overall occupancy in species within these groups, or the inability to predict occupancy from the variables included in our models. For example, floral resources and aphids are likely very important in determining Syrphidae occurrences^41^, and these variables are not included in our model.

An interesting and somewhat unexpected finding from our analysis was the negative trend we observed for land value in predicting occupancy patterns in Phoridae and Tipuloidea. The luxury effect, a well-known and studied phenomenon of many cities across the globe, suggests that wealthier areas will harbor greater biodiversity as residents of these areas have the means to support lush yards and other green amenities, plus landscaping, maintenance, and irrigation that in turn supports a high diversity of wildlife. Further, wealthy areas in arid cities, such as L.A., have far greater tree canopy cover and vegetation cover^42^, potentially creating habitats that may attract arthropods and act as a refuges in an otherwise inhospitable environment^43^. Thus, given that arthropods have particular habitat associations with various features of urban environments^23^, we expected to find positive patterns of arthropods occupancy in relation to wealth patterns. Our expectations follow those of plants^35^, birds^42^, and mammals^38^, which were all more abundant and diverse in high-income areas of cities. This raises the question of what could be driving the contrasting patterns we uncovered for arthropods (and particularly Phoridae) when compared to other taxa. A plausible explanation could be the likely increase in landscaping in high-income areas^44^, which may lead to overly manicured yards with high grass cover and high mowing intensity, each of which is negatively related to arthropod abundance and diversity. This may be particularly important in phorid flies,  where some species consume and require decaying matter in order to complete their life cycle^10^. For example, one of the phorid species that decreased significantly in occupancy with increasing land value was *Anevrina variabilis*, a species that inhabits ground squirrel burrows and feeds on decaying squirrel carcasses. Over-watering can also negatively affect habitat for phorid flies. For example, another phorid species negatively associated with land value is *Phalacrotophora halictorum*, a parasitoid of halictid bees, a group that is known to inhabit the driest areas with little or no irrigation. Further, phorid flies have been shown to be sensitive to the invasion of Argentine ants (*Linepithema humile*)^46^, as Argentine ants kill off the native ants that parasitic phorid species use as hosts.  Argentine ants have been shown to be sensitive to desiccation, and require more water availability^47^, whereas optimal conditions for many phorids are fairly dry natural areas with lots of leaf litter. Alternatively, lower-income areas may have more ‘feral’ landscaping conditions, which could lead to distinct niches that arthropods could benefit from^48^. Finally, some phorid species, such as *Megaselia scalaris* (another species whose occupancy decreased with high land values) are highly-associated with human activity, and may be more likely to occur in areas of high human density and thus low land value. Whatever the driver behind the patterns we observed for arthropods, our results suggest an extension of the luxury-effect hypothesis in that it may not apply to all arthropod groups in an urban setting. Uncovering the drivers behind this pattern could be an important avenue of future research.

Another unexpected finding was that essentially all phorid species were more likely to occur in areas further away from natural areas (Fig. 2). Much of the natural area in L.A. is concentrated in the surrounding mountains and thus tends to be at higher elevations, and remnant natural areas are very different from city gardens and parks^45^. However, this effect remained significantly negative even after accounting for the independent effect of elevation. It is possible that highly local factors are more important in shaping phorid diversity, who are (presumably) less vagile than the other groups, and thus are responding on a finer spatial scale. It is also possible that there are some fundamental differences between natural areas at different elevations in L.A. that are not captured in this analysis. For example, our definition of natural areas was quite broad and included any protected areas larger than 10 hectares. This may include heavily-modified and disturbed urban parks that may not be suitable for phorid species, but act as refuges for other arthropod species to maintain relatively high levels of diversity in urbanized landscapes (Fig. 1). Further characterization of these differences in natural area types may help unravel this relationship.

We see mixed responses both within and between groups in response to impervious surface. This was unexpected, as much research has pointed towards a negative effect of impervious surface on arthropod communities^49–51^. Often this is due to the reduction of floral resources, nesting habitat, and prey availability in these environments^31^. However, the impact of these reductions are most detrimental in specialist species who have specific life history requirements^52^, whereas generalist and highly-mobile species have the flexibility to exploit alternate resources and are less likely to be negatively impacted. Because we surveyed a broad variety of species that likely span a wide generalism-specialism spectrum, this could account for the mixed responses we see. Additionally, it is possible that impervious surface never reaches high enough levels to actually be a strongly negative effect. Recent work has hypothesized that the effects of impervious surface area don’t begin to have noticeably negative impacts on insect biodiversity until they reach more than 50% of the surrounding area^33^. Mean impervious surface area within a 0.25km radius from our study sites hovers near 55%, and so perhaps is only a significant deterrent in specialist species, and not broadly affecting most species.

Hover flies (Syrphidae) were one of the only groups to exhibit a negative response to temperature. One possible explanation could be that the food plants that maintain high aphid populations (the main food source of some syrphids^53^) dry up rapidly in the summer, particularly in areas with high mean temperature. Another possible explanation might be tied to their role as pollinators occupying a niche similar to native bees. Urban warming generally harms the abundance of native bees^33,54^, and honey bees forage more in higher temperatures and when it is sunny^55^. Considering the ubiquity of feral and managed non-native honey bees throughout our study area, we think it is plausible that the negative effect of temperature on hover fly densities is a consequence of honey bee competition. This is compatible with recent results showing a negative effect of urban honey bees on native pollinators^56^.

There are a number of caveats with our study that may influence the generalization of our findings. First, the taxonomic scope of our study is determined by the limitations imposed by our sampling method. Malaise traps were used to collect arthropods (with the exception of spiders), which trap some groups reliably, whereas some groups of arthropods are underrepresented compared to other sampling approaches^57,58^. We included all of the taxa which we had enough representation across sites and sampling months to draw reliable inferences from but had to drop others (such as Lepidoptera, Hymenoptera, etc.; see Supp. Fig. S1). Second, we collated land value data from a number of different sources and counties. Methodologies for calculating land value potentially differ across counties, and we see a noticeable jump in values at county lines (such as San Bernardino) that may be an artifact of this (Supp. Fig. S12). Third, we chose to focus solely on spatial patterns of biodiversity, and there is no temporal dimension to this study, neither seasonal nor yearly. For the vast majority of our sites, we only have 1 year of data. Any large-scale climatic anomalies could influence the composition of the arthropod communities we sampled. Arthropod communities also tend to be very stochastic and change year-to-year^59^, and we may be missing this variation. Indeed, seasonal variation in climate has been shown to be important in this system^60^, and future work will aim to decouple the spatial vs. temporal components of biodiversity in this system. Finally, Los Angeles is a unique city in some respects, particularly due to its climate (dry, hot, near-desert^23^), varied topography, and proximity to both the coast and to large protected areas. Additionally, it is distinctive in its combination of dense human population but sprawling urban footprint^61^ and the high levels of wealth disparity among its residents. However, there are many cities in the US arid west that have emulated the model of urban sprawl that Los Angeles pioneered. Therefore, we believe our results may be representative of other urban areas in western North America, though it is quite possible that we may see different trends in cities that are cooler, wetter, and have a different urbanization footprint (e.g. London or Paris).

In summary, we found that high heterogeneity in responses within and across 6 arthropod groups to (i) topographical, (ii) abiotic, and (iii) anthropogenic factors that can affect occupancy across L.A. This translates into high regional diversity and highly variable composition across space. This high variability in composition across space can have large consequences on the ecosystem services that arthropods provide across the city. Our findings are of general interest with respect to urban arthropod distributions and spatial factors that drive heterogeneity in community composition. These findings also highlight that arthropod diversity is not limited to affluent neighborhoods, and that with proper land management (such as prioritizing green areas, reducing pesticide application, and creating microhabitats in our built environments^62^), landowners can ensure that the ecosystem benefits derived from arthropods are distributed amongst all urban residents^63^. Finally, this study illustrates the unique value of community science in urban ecosystems. Not only has this project engaged Angelenos in their own backyard biodiversity and contributed to broader educational initiatives within the community^64^, it has yielded numerous novel scientific findings such as the description of species previously unknown to science^65,66^, seasonal trends in abundance^10^, and has documented major range extensions, potentially due to the introduction of non-native species^67^, all of which are relevant to site- and species-based management strategies. As such, museum-led community science initiatives like this one have an important role to play in the conservation arena^68^.

## Methods

### Community data collection

#### BioSCAN survey

BioSCAN is a community science project conducted by the Natural History Museum of Los Angeles County. The BioSCAN team recruited project participants to become BioSCAN site hosts and agreed to host arthropod sampling and environmental data collection on their properties over the course of one continuous year. All sampling took place in residential backyards and gardens in L.A. (Fig. 1). None of the sites were placed in natural areas.

Insects were sampled using a Malaise trap, a tent-like passive collection device that collects insects entering the space of the tent. Insects that are attracted to light and that move upwards when encountering obstacles will move up the mesh and fall into a sampling bottle at the top of the trap that contains ethanol to kill and preserve the specimens. Participants conducted sampling for 1 week each month for one continuous calendar year. Three distinct 1-year sampling campaigns were conducted, accumulating a total of 60 sites that were sampled for a minimum of 1 year. Each sampling campaign (phase) occurred in different years, where phase 1 occurred during 2014, phase 2 during 2015 and 2016, and phase 3 during 2017 and 2018. Staff of the museum visited each location periodically to collect accumulated insect samples and the paired environmental data (see below). For further details, see McGlynn et al.^60^.

Spiders were hand collected by the museum staff via generalized hand-collecting on the property and the general vicinity where the traps were placed.

#### Arthropod identification

BioSCAN staff and cooperating expert taxonomists identified arthropod specimens using morphology. The following groups were primarily identified to species or morphospecies level and as such are included in the analyses: Mycetophilidae, Phoridae, Drosophilidae, Tipuloidea, Syrphidae, and Araneae (Supp. Fig. S1).

### Environmental data and site characteristics

Arthropods are small-bodied ectotherms with specialized diets, so their occurrence and sampling of arthropods is a function of microhabitat characteristics. For this reason, fine-scale measurement of habitat characteristics is essential to create a predictive framework for arthropod biodiversity^69^. We used microclimate variables collected directly at the site of arthropod collections. This approach resulted in insights into the assembly of arthropod communities that would not have been possible with remote sensing or other more coarse approaches to microhabitat assessment^23,60^.

#### HOBO data loggers

Environmental data trackers were placed alongside every trap. These trackers recorded data every five minutes on air temperature, soil temperature, relative humidity, and insolation (solar radiation).

We cleaned the HOBO collected data following McGlynn et al.^60^. Specifically, we used the following steps: (i) We aggregated all site-level data into three dataframes, each corresponding to a phase of the BioSCAN data collection, removing duplicate entries and standardizing variable units across phases. For example, temperature was collected in Celsius in some phases, while in others in Fahrenheit. (ii) We filtered the variables to air temperature and relative humidity and excluded all other variables. (iii) We removed potential errors in the data. For temperature, we removed values less than $-\,5\,{^\circ }{\text{C}}$ and more than $50\,{^\circ }{\text{C}}$, and for relative humidity, we removed values less than 0% and greater than 100%. (iv) Site 07 had a sequence of months containing temperatures higher than $60\,{^\circ }{\text{C}}$, which is an unreasonably high temperature, so we removed all of this temperature data. (v) We calculated the minimum and maximum temperature (Tmin and Tmax) at the hourly scale to smooth any unreasonable temperature values captured at the 5-min period. (vi) For any hourly or daily data, we treated these data as NAN (not a number) if more than 10% of 5-min data used to aggregate was itself NAN. (vii) We then further removed observations from all sites if more than 25% of sites had NAN for that date-time combination for the variable in question. (viii) For the remaining NAN, we used inverse-distance weighting (IDW) interpolation with three nearest neighbors and a power of 1, where the neighbors are other BioSCAN HOBO sensors. IDW was performed at an hourly scale. (ix) Our annual summary statistics were then computed using this normalized, semi-gap-filled data.

Using the site-level micro-meteorological data from the HOBO data loggers, we calculated several bioclimatic variables at an annual scale^70^ to obtain environmental metrics for each site that encompassed temporal variation. This way, landscape and site environmental characteristics were comparable (i.e., accounting spatial variation) and occupancy estimates could be interpolated across L.A. From the available suite of bioclimatic variables we used only mean annual temperature (BIO1) and mean diurnal range (BIO2) in our occupancy models.

#### Meteorological data

We downloaded Bioclimatic variables from WorldClim^70^ to create the interpolated map of species richness for L.A. (see Spatial Interpolation).

#### Land value

Land parcel data, including parcel delineation, land value, use type, and year assessed, was available and downloaded for Los Angeles and San Bernardino counties^71,72^. We filtered for residential parcels only and scaled parcel values to the estimated current value using historic Consumer Price Index estimates. Parcel values were then normalized by area and unreasonably low values were filtered from the dataset.

Using this standardized parcel dataset, we calculated neighborhood-level land value estimates for each of the BioSCAN sites by taking the mean land value of residential parcels within two buffer sizes around each sampling site: 0.25 km and 0.5 km. We chose to calculate these buffers at different scales because of the differing dispersal abilities between the taxonomic groups included in our study. While some groups (e.g. Syrphids) can disperse relatively far^73^, and will likely be influenced by the habitat within a a radius of 0.5–1 km^73^, other groups (e.g. Phorids) are known to not disperse very far at all^74^ (and sometimes less than 30 m^75^), and so habitat beyond 0.25 km is likely not relevant for these groups. Land value buffers were set at 0.25 km for Phoridae, Araneae; and 0.5 km for Syrphidae, Tipuloidea, Drosophilidae, and Mycetophilidae.

#### Impervious surface

We calculated neighborhood-level impervious surface percentage estimates in a similar way to land value. We downloaded 2019 National Land Cover Database (NLCD) Impervious Surface cover data^76^ at the 30 m resolution for L.A. and the mean impervious cover percentage value was calculated within two buffer sizes around each sampling site: 0.25 km, and 0.5 km. The same buffer sizes as from land value were assigned to each taxonomic group.

#### Elevation

We downloaded and extracted elevation data at the 30 m resolution using the void-filled NASADEM product^77^.

#### Distance to ocean

Sites in our study vary in their proximity to the Pacific Ocean and topological features. Proximity to the ocean mediates many of the meteorological effects that differentiate coastal and inland areas, such as solar radiation, weather systems, and the distribution of moisture. Additionally, meteorological conditions do not change linearly with geographic distance; they are mediated by topological features, such as mountains (e.g. rain shadows). As such, we developed a cost distance to ocean map for L.A. We created a cost distance raster using the cost distance tool in ArcGIS^78^ where a polygon of the Pacific Ocean was the source layer and slope was the cost (derived from the NASADEM elevation data). Here, slope acts as a surrogate for topological features that disrupt meteorological conditions, namely mountains.

#### Distance to natural areas

Some parks and protected areas contain native habitat and complex vegetation structures not commonly found across urbanized L.A., potentially resulting in different community structures in neighboring areas. Additionally, natural areas are thought to be important reservoirs of source populations for insects^79^, and so sites closer to natural areas may experience more frequent colonization events. We used the sf package^80^ in R^81^ to calculate a raster with cell values corresponding to the distance to nearest protected area. We used the California Protected Area Database^82^ as a surrogate for natural areas, removing protected areas less than 10 hectares in size to remove small, manicured parks that may not provide the habitat characteristics that larger, more natural areas do.

### Causal analysis

To estimate the effect sizes of different variables of interest (mean annual air temperature, mean diurnal range, land value, percentage impervious cover, distance to natural areas, and distance to ocean) on occupancy, we use a structural causal modeling framework^83^. For each group studied, we developed a directed acyclic graph (DAG) based on shared life history characteristics of the group and probable influential variables related to our research question (Supp. Figs. S3–S5). Visualizations of each DAG were produced using the R packages daggity^84^ and ggplot2^85^. The relationships we established in the DAGs were dependent on the climatic and social relationships we find in the greater L.A. metro area. For example, in L.A., we observe that higher elevation areas or areas closer to the ocean have higher living desirability.

After developing a DAG for each taxonomic group studied, we followed the structural causal modelling framework described by Arif and MacNeil 2022^83^ to validate our DAGs with observed variable data and apply backdoor criterion in order to select the appropriate controlling variables for each regression model. The R package daggity^84^ was used once more to test DAG and data consistency among observed variables that could not be explained by the relationships specified. After correcting the DAGs for unexplained correlations, we applied the backdoor criterion to select a single model for each variable of interest to estimate the variable’s effect on occupancy.

### Occupancy models

We used an occupancy modeling approach to evaluate the effect of the different predictors for each arthropod group. We ran a multi-species occupancy model for each group (Mycetophilidae, Phoridae, Drosophilidae, Tipuloidea, Syrphidae, Araneae). Following Stewart et al.^25^, we predicted occupancy probability using an information-theoretic approach using all observed variables but used a structural causal modeling framework approach to estimate and compare the effects of each observed variable on arthropod occupancy using separate models informed by our DAGs. Because of this, we ran a total of 37 occupancy models (6 arthropod groups $\times$ 6 environmental predictors of interest, plus an additional environmental predictor for Phoridae only). Observed variables include site averaged annual mean temperature, site averaged mean diurnal range, site averaged relative humidity, site elevation, site distance to ocean, site distance to natural areas, neighbourhood level impervious surface, and neighbourhood level land value. We ran the following models.

*Model all:* is the occupancy model that we developed to predict arthropod occupancy across L.A.

1 $$\begin{aligned} \begin{aligned} \text {logit}(\psi _{ij}) =&\psi _0 + \\&\psi _{\textrm{Species}}[i] + \\&\psi _{\mathrm {T_{Mean}}}[i] \times \mathrm {T_{Mean}}[j] +\\&\psi _{\mathrm {T_{Diurnal Range}}}[i] \times \mathrm {T_{Diurnal Range}}[j] +\\&\psi _{\textrm{Elevation}}[i] \times \textrm{Elevation}[j] +\\&\psi _{\mathrm {D_{Ocean}}}[i] \times \mathrm {D_{Ocean}}[j] +\\&\psi _{\mathrm {D_{NaturalAreas}}}[i] \times \mathrm {D_{NaturalAreas}}[j] +\\&\psi _{\textrm{ImperviousSurface}}[i] \times \textrm{ImperviousSurface}[j] +\\&\psi _{\textrm{LandValue}}[i]\times \textrm{LandValue}[j]. \\ \\ \end{aligned} \end{aligned}$$

We estimated occupancy for each species *i* in each site *j*, where $\psi _0$ and, $\psi _{\textrm{Species}}[i]$ are defined as a global intercept and a species-specific intercept, respectively. $\psi _{\mathrm {T_{Mean}}}[i]$, $\psi _{\mathrm {T_{Diurnal Range}}}[i]$, $\psi _{\textrm{Elevation}}[i]$, $\psi _{\mathrm {D_{Ocean}}}[i]$, $\psi _{\mathrm {D_{NaturalAreas}}}[i]$, $\psi _{\textrm{ImperviousSurface}}[i]$, and $\psi _{\textrm{LandValue}}[i]$ are species-specific linear effects of mean annual temperature, mean annual diurnal range, elevation, site distance to the ocean, site distance to natural areas, neighborhood impervious surface, and neighborhood land value, respectively.

*Model distance to ocean* is the occupancy model that we used to estimate the effect of distance to the ocean on occupancy.

2 $$\begin{aligned} \begin{aligned} \text {logit}(\psi _{ij}) =&\psi _0 + \\&\psi _{\textrm{Species}}[i] + \\&\psi _{\mathrm {D_{Ocean}}}[i] \times \mathrm {D_{Ocean}}[j] +.\\ \\ \end{aligned} \end{aligned}$$

*Model distance to natural areas* is the occupancy model that we used to estimate the effect of distance to natural areas on occupancy.

3 $$\begin{aligned} \begin{aligned} \text {logit}(\psi _{ij}) =&\psi _0 + \\&\psi _{\textrm{Species}}[i] + \\&\psi _{\mathrm {D_{NaturalAreas}}}[i] \times \mathrm {D_{NaturalAreas}}[j] +\\&\psi _{\textrm{Elevation}}[i] \times \textrm{Elevation}[j]. \\ \\ \end{aligned} \end{aligned}$$

*Model mean annual temperature* is the occupancy model that we used to estimate the effect of mean annual temperature on occupancy.

4 $$\begin{aligned} \begin{aligned} \text {logit}(\psi _{ij}) =&\psi _0 + \\&\psi _{\textrm{Species}}[i] + \\&\psi _{\mathrm {T_{Mean}}}[i] \times \mathrm {T_{Mean}}[j] +.\\ \\ \end{aligned} \end{aligned}$$

*Model Diurnal range* is the occupancy model that we use to estimate the effect of the Diurnal range on occupancy.

5 $$\begin{aligned} \begin{aligned} \text {logit}(\psi _{ij}) =&\psi _0 + \\&\psi _{\textrm{Species}}[i] + \\&\psi _{\mathrm {T_{Diurnal Range}}}[i] \times \mathrm {T_{Diurnal Range}}[j] +\\&\psi _{\mathrm {D_{Ocean}}}[i] \times \mathrm {D_{Ocean}}[j]. \\ \end{aligned} \end{aligned}$$

*Model relative humidity* is the occupancy model that we use to estimate the effect of the Relative humidity on occupancy. This model was run on Phoridae only.

6 $$\begin{aligned} \begin{aligned} \text {logit}(\psi _{ij}) =&\psi _0 + \\&\psi _{\textrm{Species}}[i] + \\&\psi _{\mathrm {T_{Mean}}}[i] \times \mathrm {T_{Mean}}[j] +\\&\psi _{\mathrm {RH_{Mean}}}[i] \times \mathrm {RH_{Mean}}[j] +\\&\psi _{\textrm{ImperviousSurface}}[i] \times \textrm{ImperviousSurface}[j] +\\&\psi _{\mathrm {D_{Ocean}}}[i] \times \mathrm {D_{Ocean}}[j] +\\&\psi _{\textrm{Elevation}}[i] \times \textrm{Elevation}[j]. \\ \end{aligned} \end{aligned}$$

*Model impervious surface and land value* is the occupancy model that we used to estimate the effect of impervious surface and land value on occupancy.

7 $$\begin{aligned} \begin{aligned} \text {logit}(\psi _{ij}) =&\psi _0 + \\&\psi _{\textrm{Species}}[i] + \\&\psi _{\textrm{Species}}[i] + \\&\psi _{\textrm{Elevation}}[i] \times \textrm{Elevation}[j] +\\&\psi _{\mathrm {D_{Ocean}}}[i] \times \mathrm {D_{Ocean}}[j] +\\&\psi _{\mathrm {D_{NaturalAreas}}}[i] \times \mathrm {D_{NaturalAreas}}[j] +\\&\psi _{\textrm{ImperviousSurface}}[i] \times \textrm{ImperviousSurface}[j] +\\&\psi _{\textrm{LandValue}}[i]\times \textrm{LandValue}[j]. \\ \\ \end{aligned} \end{aligned}$$

For all models, we assume that species-specific intercepts and slopes in both of the above models are normally distributed about some mean. Specifically,

8 $$\begin{aligned} \begin{aligned} \psi _{\textrm{Species}}[i]&\sim {\mathcal {N}}(0, \sigma _{\psi \textrm{Species}})\\ \psi _{\mathrm {T_{Mean}}}[i]&\sim {\mathcal {N}}(\mu _{\psi \mathrm {T_{Mean}}}, \sigma _{\psi \mathrm {T_{Mean}}})\\ \psi _{\mathrm {T_{Diurnal Range}}}[i]&\sim {\mathcal {N}}(\mu _{\psi \mathrm {T_{Diurnal Range}}}, \sigma _{\psi \mathrm {T_{Diurnal Range}}})\\ \psi _{\mathrm {RH_{Mean}}}[i]&\sim {\mathcal {N}}(\mu _{\psi \mathrm {RH_{Mean}}}, \sigma _{\psi \mathrm {RH_{Mean}}})\\ \psi _{\textrm{Elevation}}[i]&\sim {\mathcal {N}}(\mu _{\psi \textrm{Elevation}}, \sigma _{\psi \textrm{Elevation}})\\ \psi _{\mathrm {D_{Ocean}}}[i]&\sim {\mathcal {N}}(\mu _{\psi \mathrm {D_{Ocean}}}, \sigma _{\psi \mathrm {D_{Ocean}}})\\ \psi _{\mathrm {D_{NaturalAreas}}}[i]&\sim {\mathcal {N}}(\mu _{\psi \mathrm {D_{NaturalAreas}}}, \sigma _{\psi \mathrm {D_{NaturalAreas}}})\\ \psi _{\textrm{ImperviousSurface}}[i]&\sim {\mathcal {N}}(\mu _{\psi \textrm{ImperviousSurface}}, \sigma _{\psi \textrm{ImperviousSurface}})\\ \psi _{\textrm{LandValue}}[i]&\sim {\mathcal {N}}(\mu _{\psi \textrm{LandValue}}, \sigma _{\psi \textrm{LandValue}}),\hspace{0.1ex}\\ \end{aligned} \end{aligned}$$

where $\mu _{\psi \mathrm {T_{Mean}}}$, $\mu _{\psi \mathrm {T_{Diurnal Range}}}$, $\mu _{\psi \textrm{Elevation}}$, $\mu _{\psi \mathrm {D_{Ocean}}}$, $\mu _{\psi \mathrm {D_{NaturalAreas}}}$, $\mu _{\psi \textrm{ImperviousSurface}}$, $\mu _{\psi \textrm{LandValue}}$ denote the mean effect of each corresponding predictor, across species, and $\sigma$ terms denote the variances about these means.

In both of the above models, we model detection probability as

9 $$\begin{aligned} \begin{aligned} \text {logit}(p_{ijk}) =&p_0 + \\&p_{\mathrm {Month^2}} \times \mathrm {Month^2}[i] +\\&p_{\textrm{Species}}[i], \\ \end{aligned} \end{aligned}$$

where $p_0$ denotes the mean detection probability and $p_{\textrm{Species}}[i]$ denotes a species-specific random effect. While $p_{\mathrm {Month^2}}$ are fixed effects that change as a function of the month. The fixed effect of month assumes detection is highest during the middle of the year and decreases towards the beginning and end of the year.

Additionally, we assume

10 $$\begin{aligned} p_{\textrm{Species}}[i] \sim {\mathcal {N}}(0, \sigma _{p\textrm{Species}}).\hspace{0.1ex} \end{aligned}$$

We ran occupancy models in JAGS^86^ and assessed model convergence both by visually inspecting chains and checking The Gelman–Rubin statistic (we ensured that ${\hat{R}}$ was $< 1.1$ for all parameters). We used flat, uninformative priors for all parameters and ran models for 100,000 iterations, with a burn-in of 1000 iterations and thinning every 100 iterations, across 3 chains, resulting in a total of 300 posterior samples.

### Spatial interpolation of results

We estimated species richness for our study groups across L.A. by spatially interpolating our linear occupancy model, fit with species-specific mean posterior effect sizes for each predictor and intercept. Input spatial data was consistent with the data used for occupancy models with the exception of the two bioclimatic variables: mean annual air temperature (BIO1) and mean diurnal range (BIO2). The HOBO temperature data is extremely fine-scale in its temporal and spatial resolution and so will be the most accurate data in predicting species-specific occupancy at each site. However, the HOBO temperature data is very limited in spatial extent (we only had data loggers at the 60 sites) so interpolating site-level temperature values across the remainder of the LA Basin would be unfeasible. For this reason, we used WorldClim^70^ data to interpolate site-level climate across the basin.

Data processing to produce these maps included the following. (i) We aligned and projected all spatial predictors to the WGS 84 projection system, masked and snapped to a common configuration, and assigned the same cell size. (ii) We scaled and log-transformed input data, as was done to prepare the data for the occupancy model. We also re-centered mean annual air temperature and mean diurnal range estimates due to varying sampling-site mean estimates (as a result of the different data sources). Here, we assume that the BioCLIM variables scale linearly with site-level difference observations. (iii) We filtered values of each predictor if it was beyond three standard deviations about the mean of our model inputs (zero after scaling); values beyond this were set to Not-a-number (NAN). (iv) For each 1-km grid cell across L.A., if any predictor had a value of NAN, we set to NAN to normalize the extent, thus generating the extent seen in Fig. 1.

After processing the spatial data, we calculated species-specific occupancy posterior probabilities for each 1-km cell using the *Model All*. We then summed the median posterior occupancy for each species at each cell for all species, first according to group, then collectively for all groups, to obtain richness estimates (Fig. 1).

For most analyses we used R V4.2.1^81^. For spatial manipulations we used the packages sf^80^ and raster^87^; for data manipulation and visualization we used tidyverse^88^; for running models, we used rjags^89^, R2jags^90^, and runjags^91^. For manipulation of the environmental data we used Python V3.8.9^92^, and for some spatial analyses we used ArcGIS Pro V3.0^78^. To access and download the spatial data used, we used Google Earth Engine^93^. To generate maps for Fig. 1, Figs. S2, S12–S17, we used the packages ggplot2^85^ and ggmap^94^ for visualization, and basemaps^95^ for downloading the basemap, which used the toner-lite map tiles by Stamen Design, under CC BY 4.0. Data by OpenStreetMap, under ODbL.

### Supplementary Information

Supplementary Figures.

## Data Availability

All code used to produce these results is freely available in a public GitHub repository (https://github.com/EcolDataSciUSC/BioSCAN_2023.git). The repository includes most external data—as well as the steps to access and download it—and BioSCAN collection data can be obtained by contacting the corresponding author.
