# Supplementary material for: Drivers of arthropod biodiversity in an urban ecosystem
Source: Sci Rep. 2024 Jan 3;14:390. doi: 10.1038/s41598-023-50675-3 (PMC10764344; doi:10.1038/s41598-023-50675-3)
Supplement: Supplementary file 1 — Supplementary Figures. [file 41598_2023_50675_MOESM1_ESM.pdf]

Supplementary Material

Species Identification: Figure S1
Width of Credible Interval for Richness Map Estimates: Figure S2
DAGS: Figures S3, S4, S5
Relative Humidity: Figure S6
Spaghetti plots of other insect groups: Figures S7, S8, S9, S10, S11
Maps of Predictor Values: Figures S12, S13, S14, S15, S16, S17

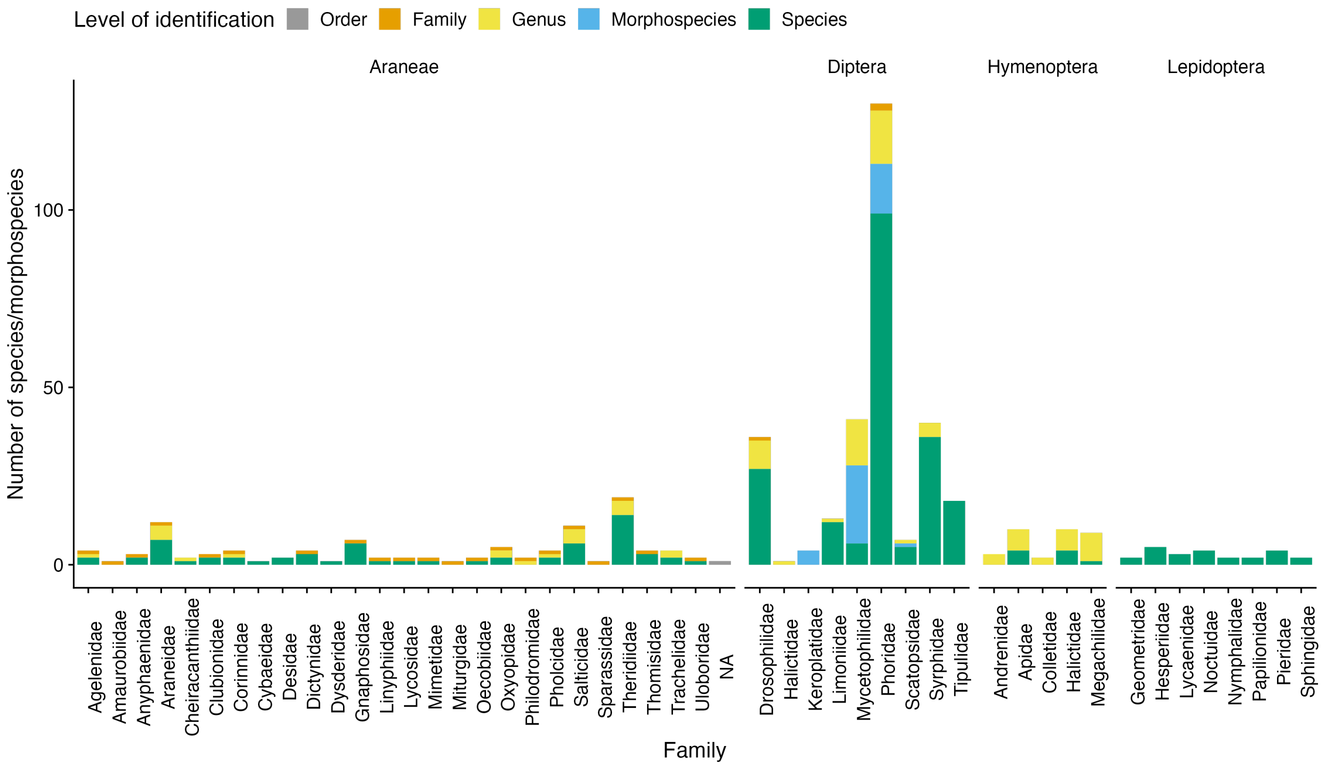

Figure S1. Precision of taxonomic identification, sorted by multiple taxonomic scales including family and order. The majority of individuals were identified to the species level, but it is apparent there is identification bias in this dataset, as some families are much more precisely identified than other, with orders like Hymenoptera being primarily identified only to the genus level.

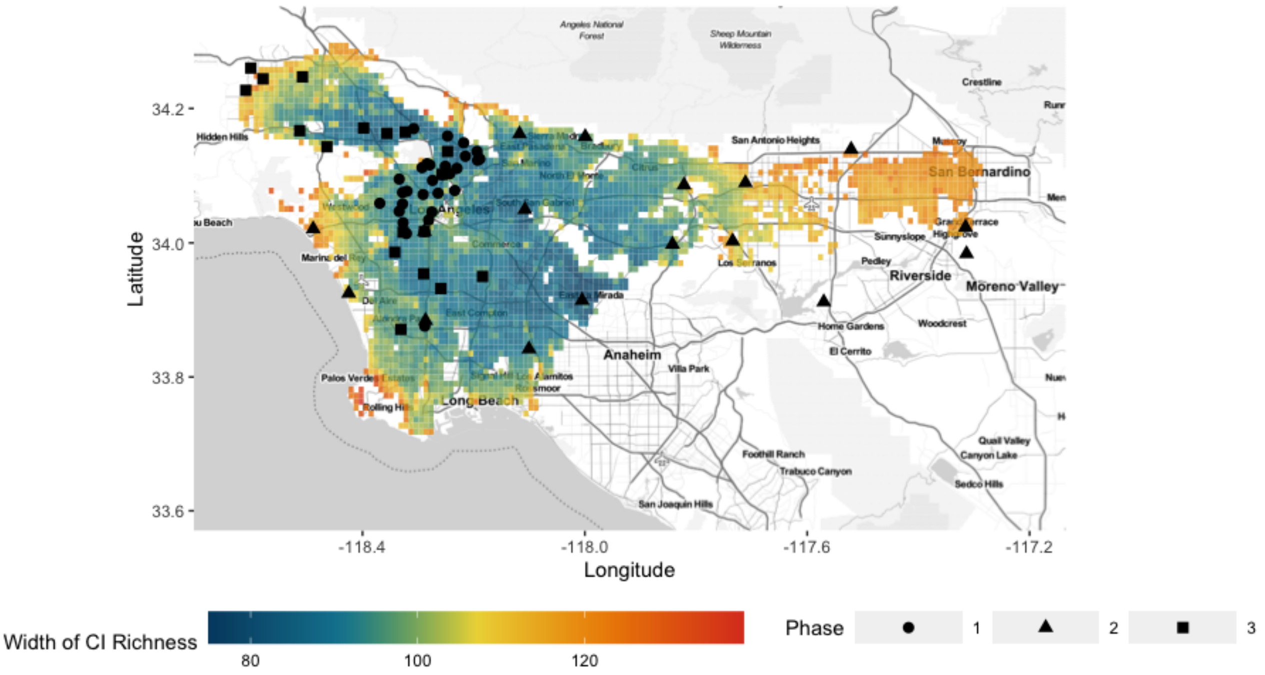

Figure S2. Width of Figure 1a credible interval (CI). Like the richness estimates shown in Figure 1a, chains were used to compute occupancy probabilities for every iteration and for every species. Here, the 2.5% and 97.5% quantiles of occupancy probabilities were pulled for each species in each cell and the difference between the two was calculated to yield the CI width. For example, if a cell has a predicted richness of 100 and a CI width of 50, the actual richness is likely between 75 and 125, plus or minus some skew in the distribution. R packages ggplot2, ggmap, and basemaps were used to create this map. Map tiles by Stamen Design, under CC BY 4.0. Data by OpenStreetMap, under ODbL.

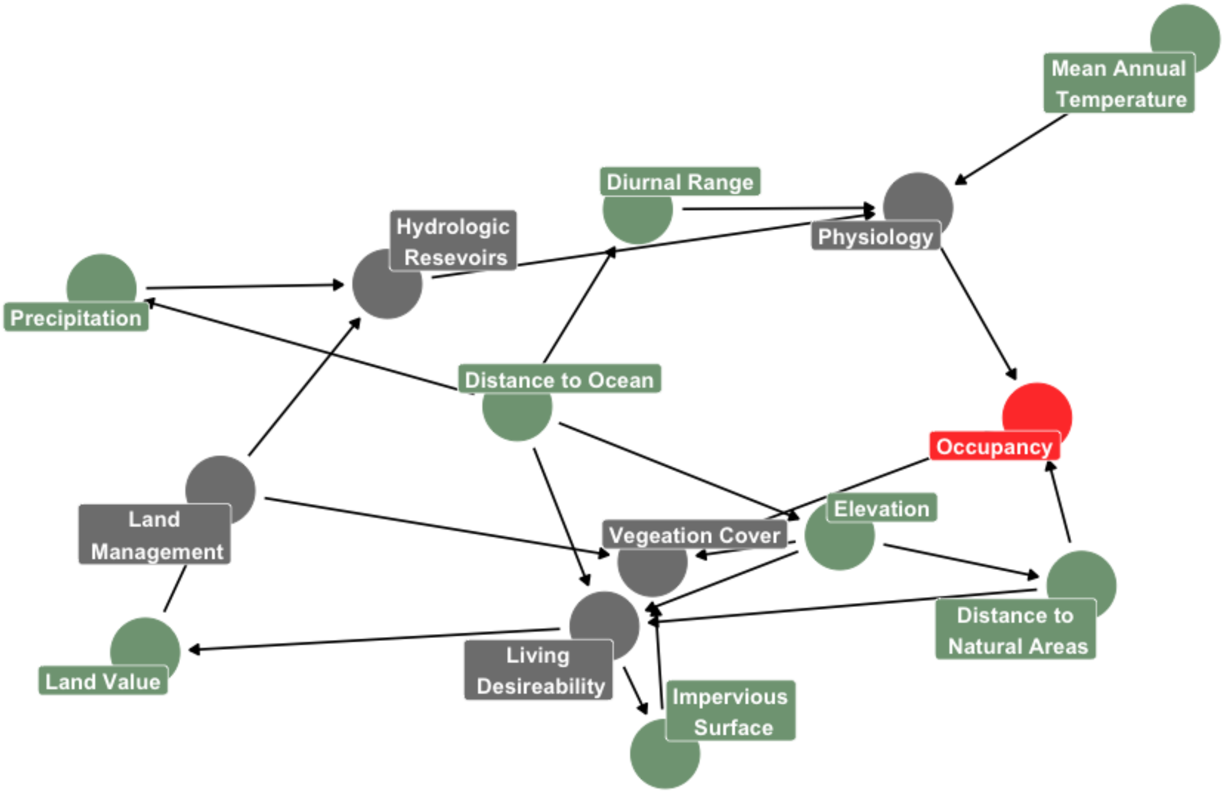

Figure S3. Directed Acyclic Diagram (DAG) for Tipuloidea, generated using the daggity and ggplot2 R packages. Our measured variables are included in each group's DAG, with causal effects based on physiology/general life history of the group and effects of additional latent variables. Here, hydrologic reservoirs are included since tipulids generally require water bodies to complete their life cycle. We assume occupancy is governed directly by three variables: distance to natural areas, physiology, and vegetation cover.

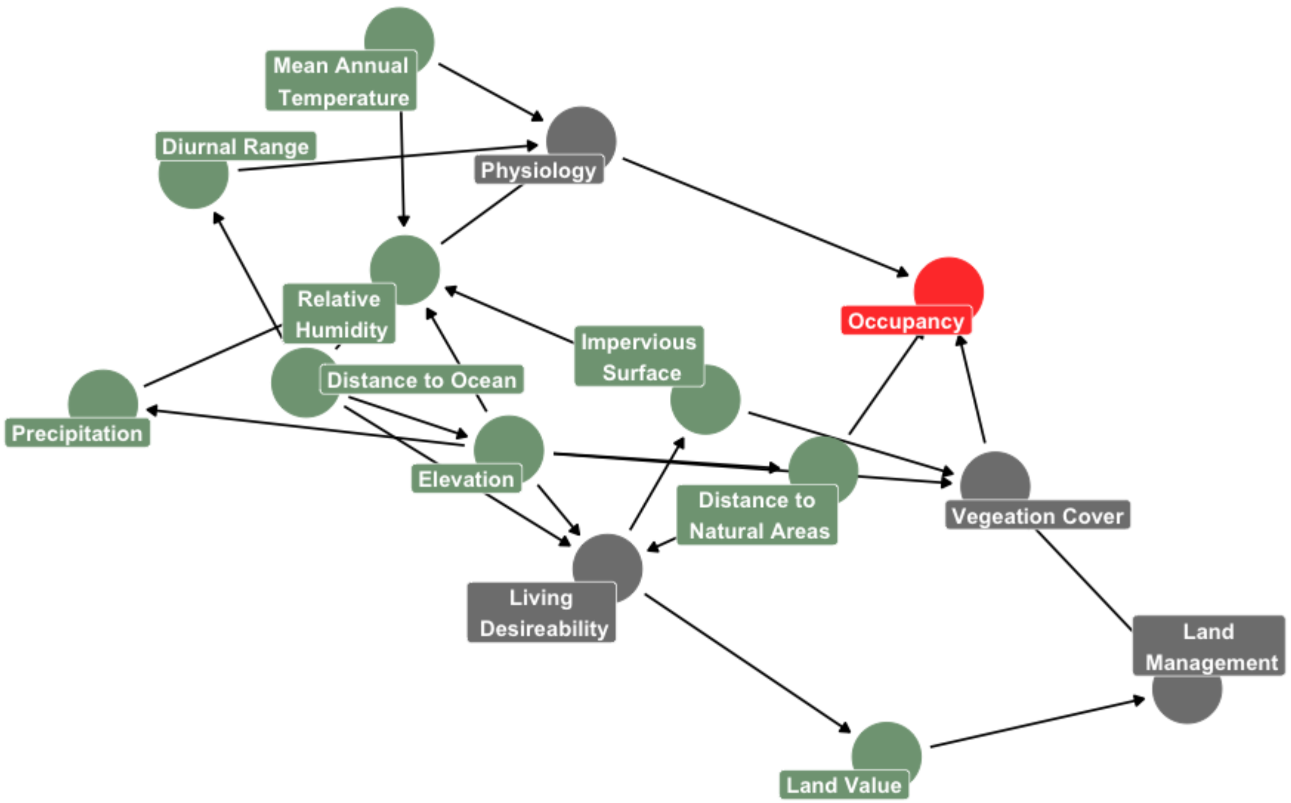

Figure S4. Directed Acyclic Diagram (DAG) for Phoridae, generated using the daggity and ggplot2 R packages. Our measured variables are included in each group's DAG, with causal effects based on physiology/general life history of the group and effects of additional latent variables. Here, relative humidity is included as a measured variable since it can be an important requirement for phorid life cycles and foraging behaviour. We assume occupancy is governed directly by three variables: distance to natural areas, physiology, and vegetation cover.

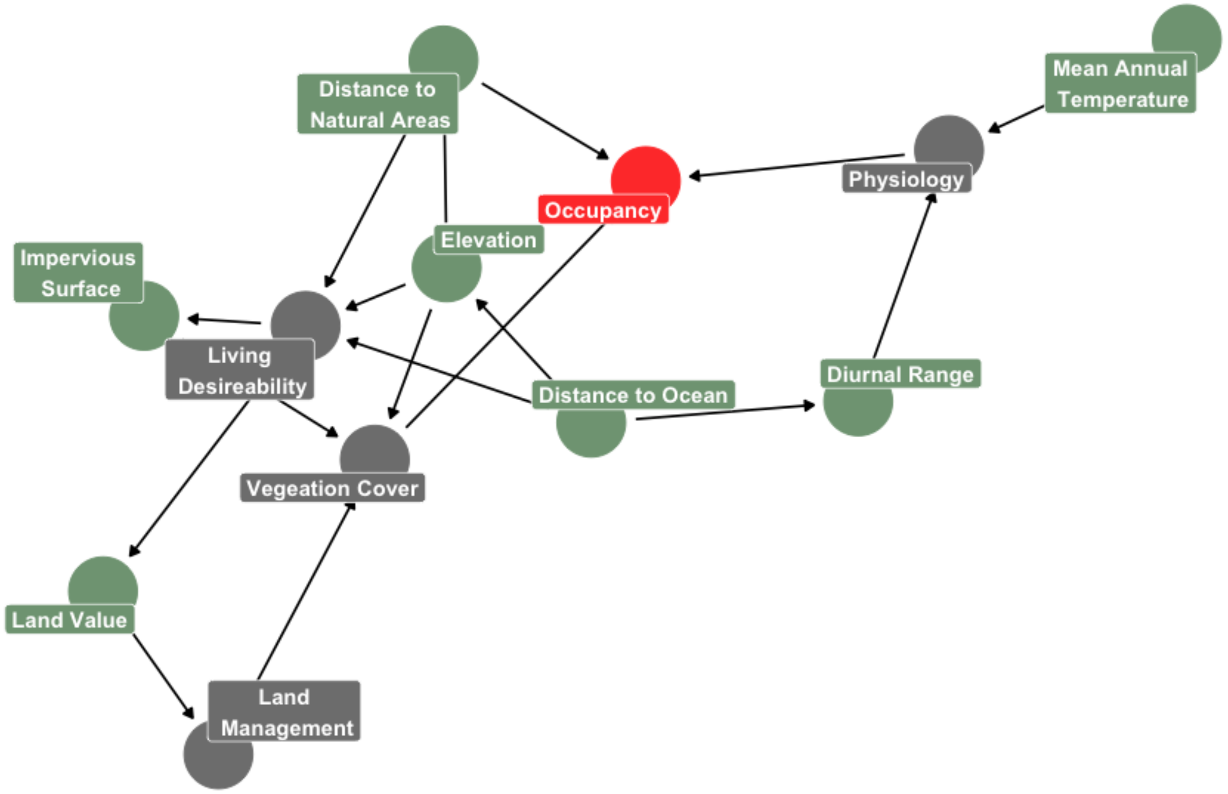

Figure S5. Directed Acyclic Diagram (DAG) for remaining groups (Drosophilidae, Mycetophilidae, Syrphidae, and Araneae), generated using the daggity and ggplot2 R packages. Our measured variables are included in each group's DAG, with causal effects based on physiology/general life history of the group and effects of additional latent variables. We assume occupancy is governed directly by three variables: distance to natural areas, physiology, and vegetation cover.

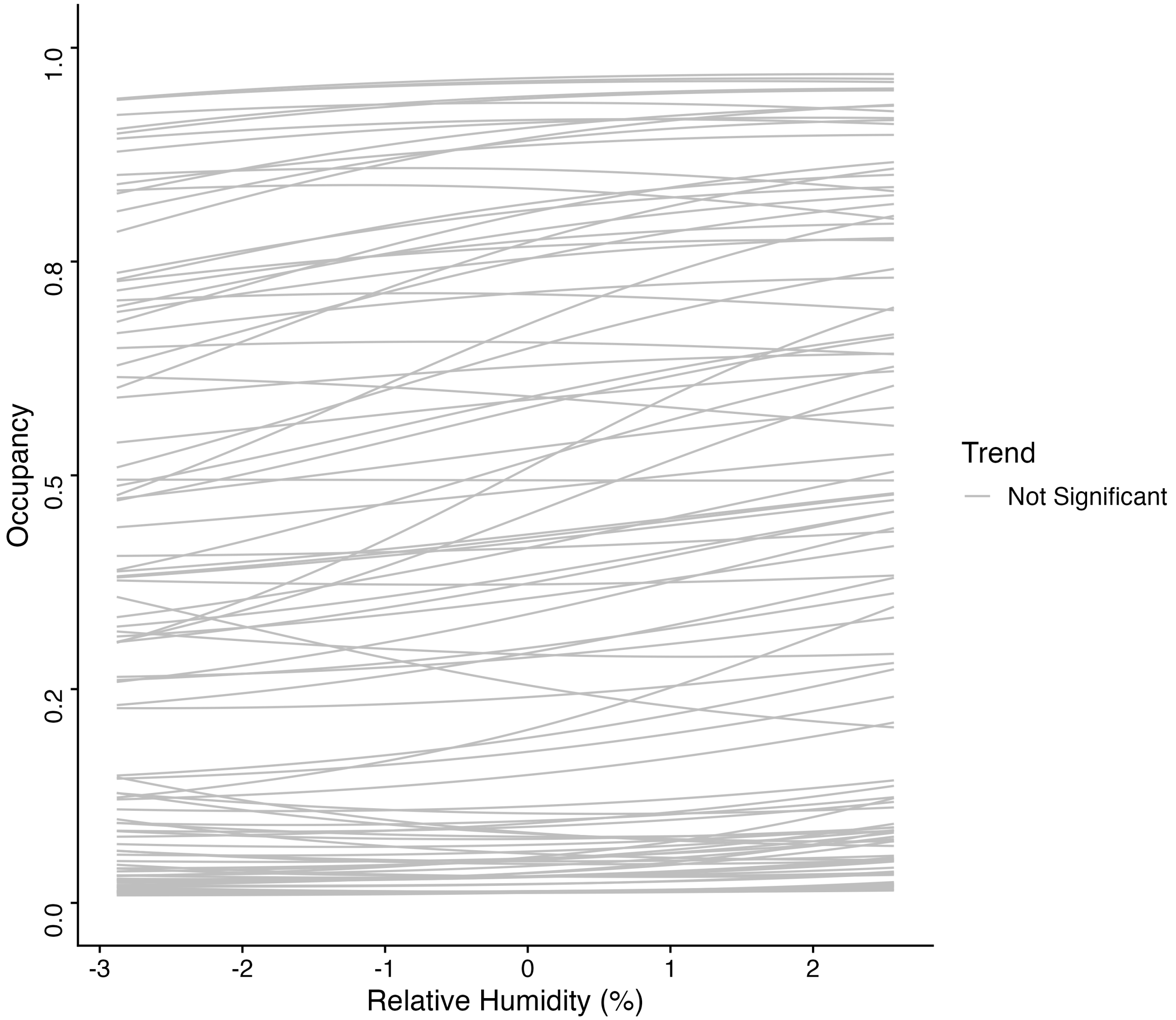

Figure S6. Within-group predictors of occupancy in Phoridae. 108 species are present in this group. This plot is showing the single effect of relative humidity by keeping the other environmental variables in the same model at their average value from the sampling data. Each line represents the effect of the environmental variable on an individual species. The color of the line represents a negative (red), not significant (grey), or positive (blue) correlation between the occupancy of a species and an environmental variable.

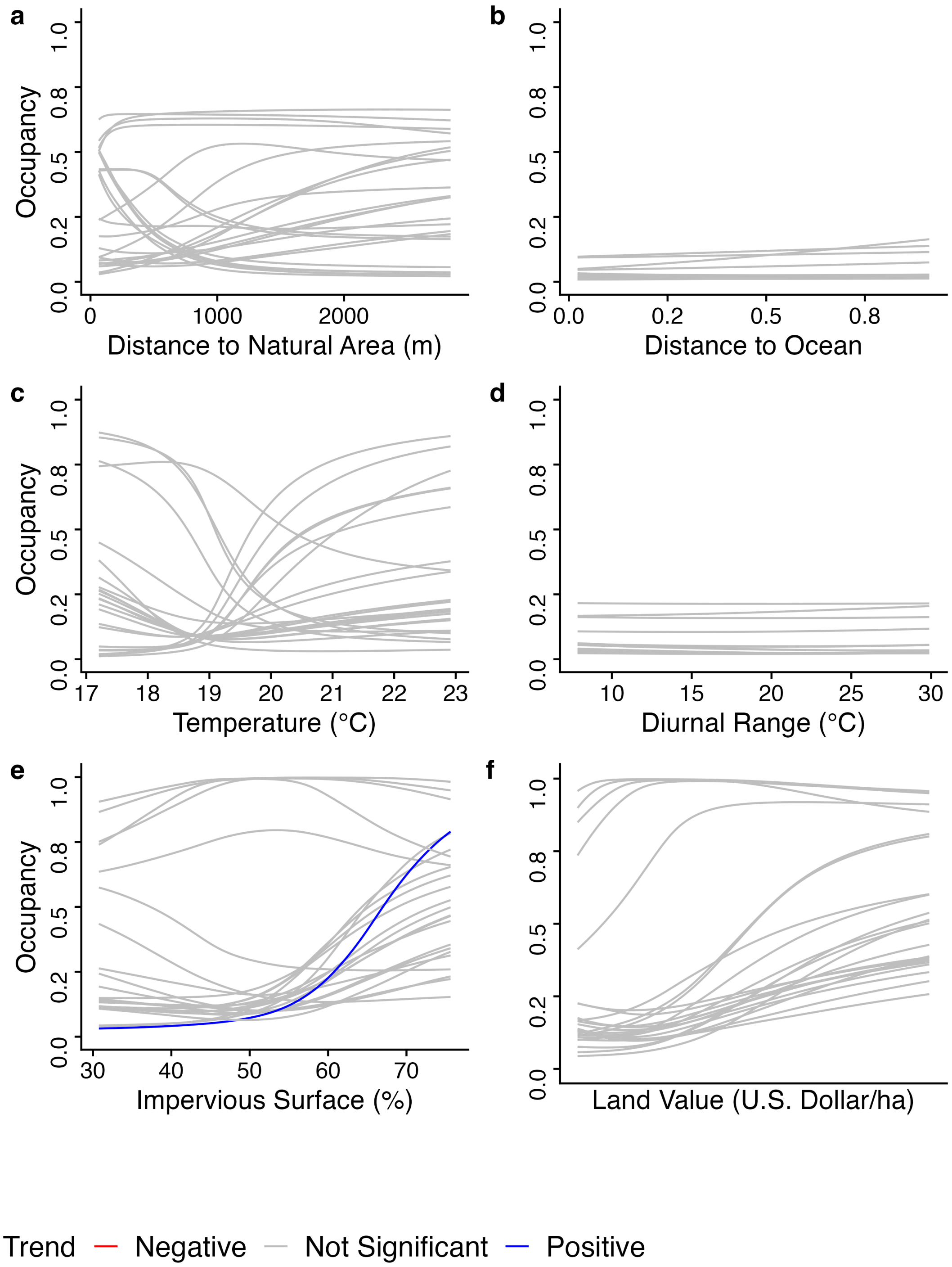

Figure S7. Within-group predictors of occupancy in Araneae. 24 species are present in this group. Each plot is showing the single effect of an environmental variable by keeping the other environmental variables in the same model at their average value from the sampling data. Each line represents the effect of the environmental variable on an individual species. The color of the line represents a negative (red), not significant (grey), or positive (blue) correlation between the occupancy of a species and an environmental variable.

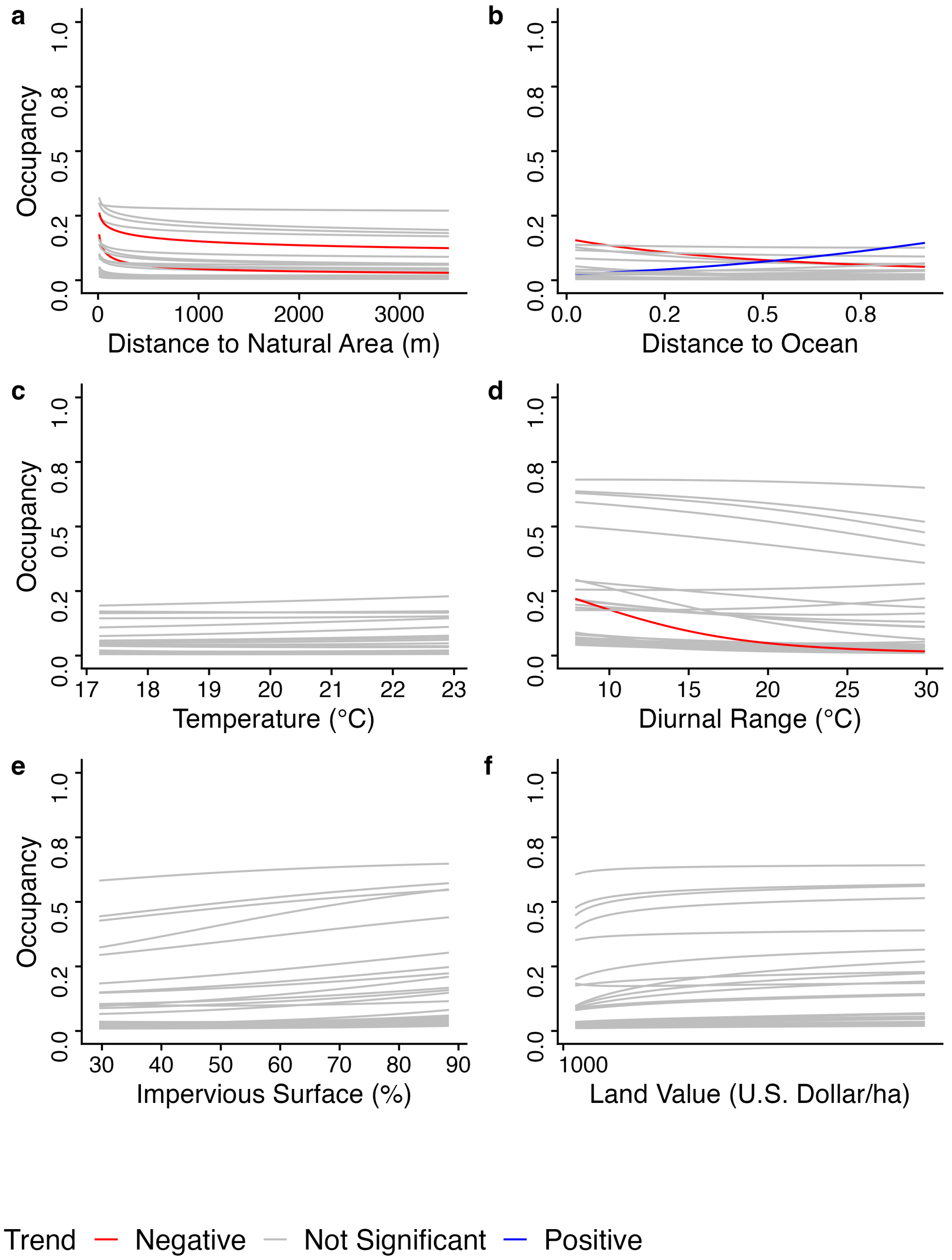

Figure S8. Within-group predictors of occupancy in Drosophilidae. 27 species are present in this group. Each plot is showing the single effect of an environmental variable by keeping the other environmental variables in the same model at their average value from the sampling data. Each line represents the effect of the environmental variable on an individual species. The color of the line represents a negative (red), not significant (grey), or positive (blue) correlation between the occupancy of a species and an environmental variable.

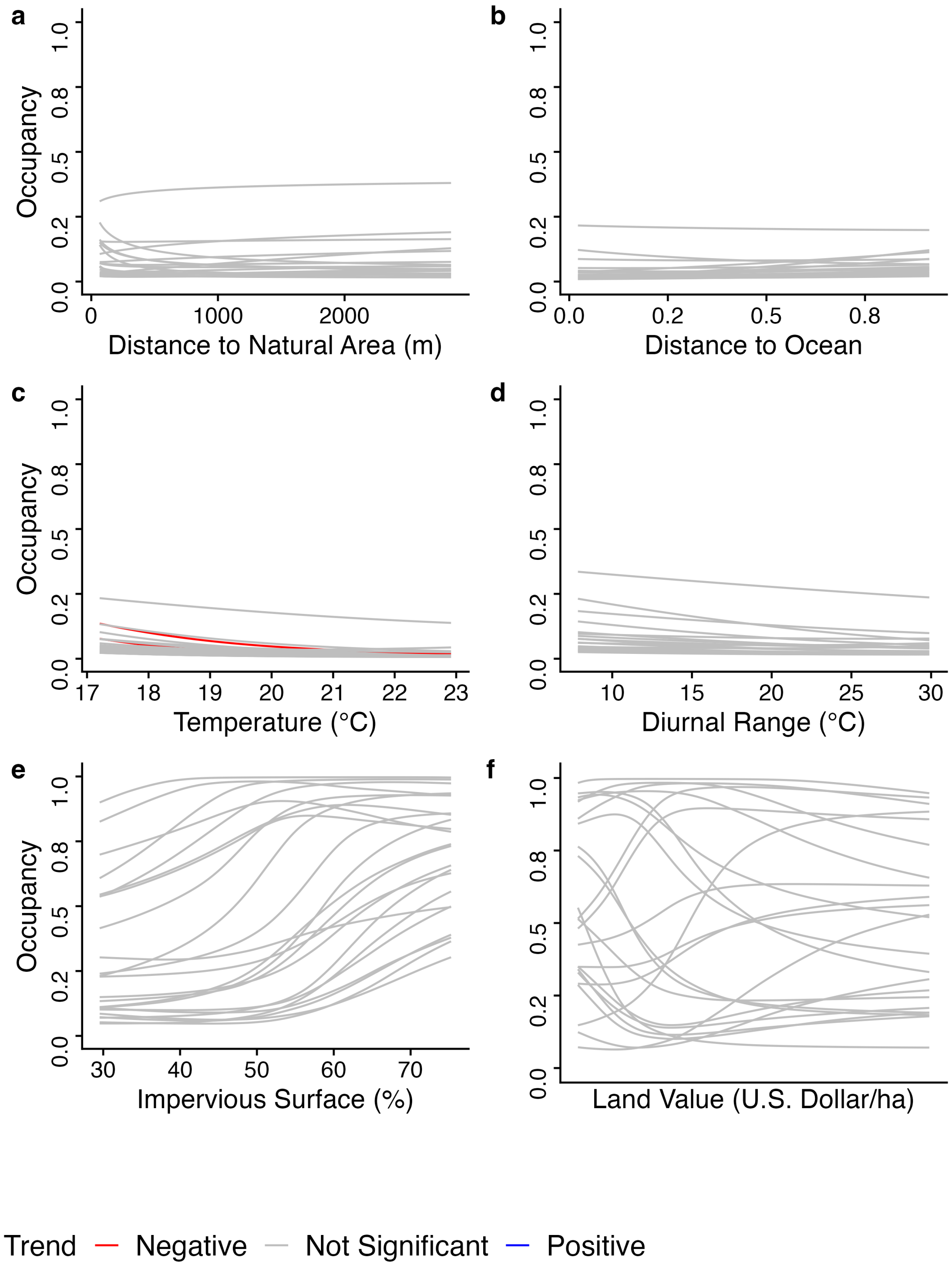

Figure S9. Within-group predictors of occupancy in Mycetophilidae. 23 species are present in this group. Each plot is showing the single effect of an environmental variable by keeping the other environmental variables in the same model at their average value from the sampling data. Each line represents the effect of the environmental variable on an individual species. The color of the line represents a negative (red), not significant (grey), or positive (blue) correlation between the occupancy of a species and an environmental variable.

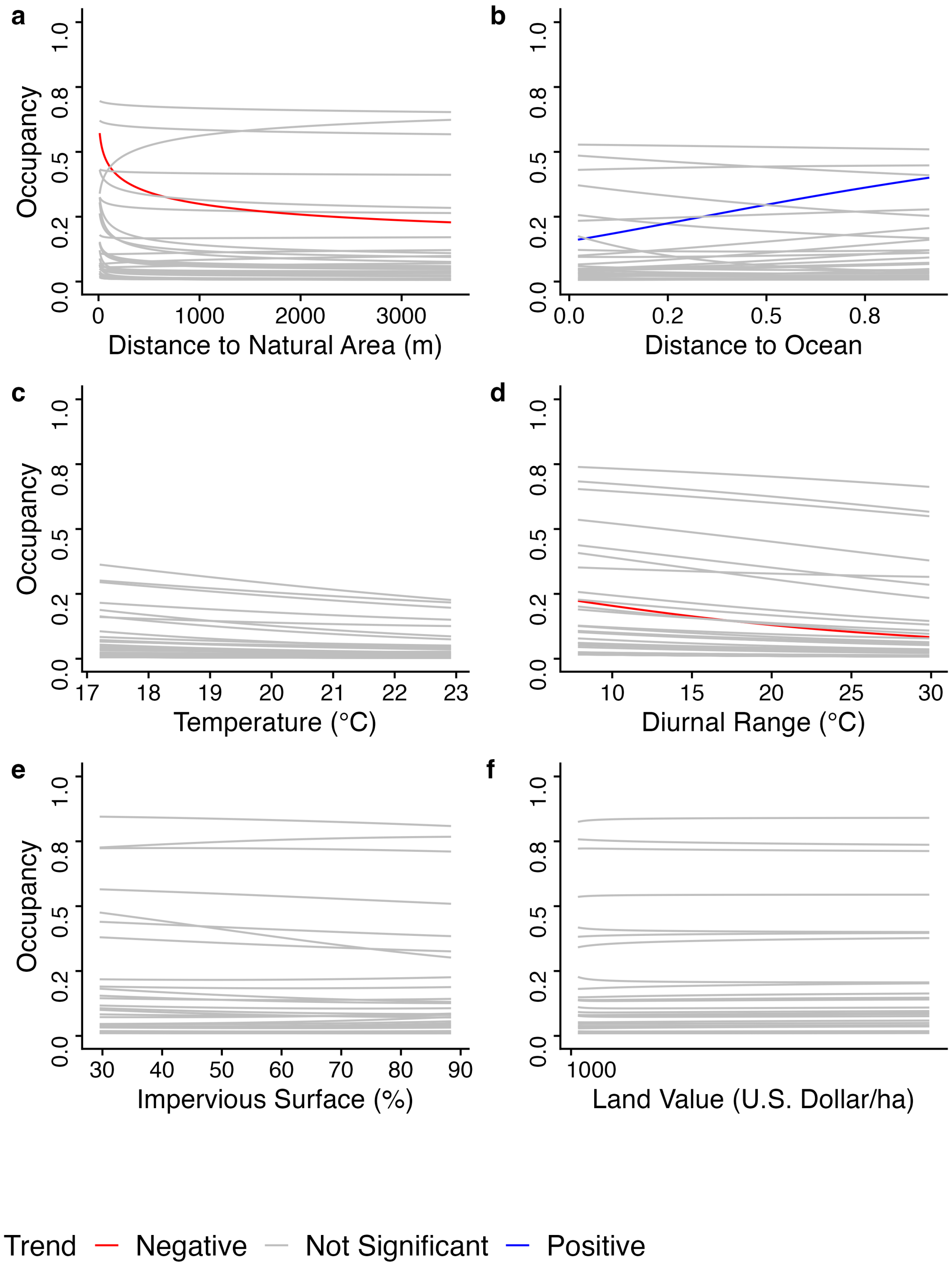

Figure S10. Within-group predictors of occupancy in Syrphidae. 34 species are present in this group. Each plot is showing the single effect of an environmental variable by keeping the other environmental variables in the same model at their average value from the sampling data. Each line represents the effect of the environmental variable on an individual species. The color of the line represents a negative (red), not significant (grey), or positive (blue) correlation between the occupancy of a species and an environmental variable.

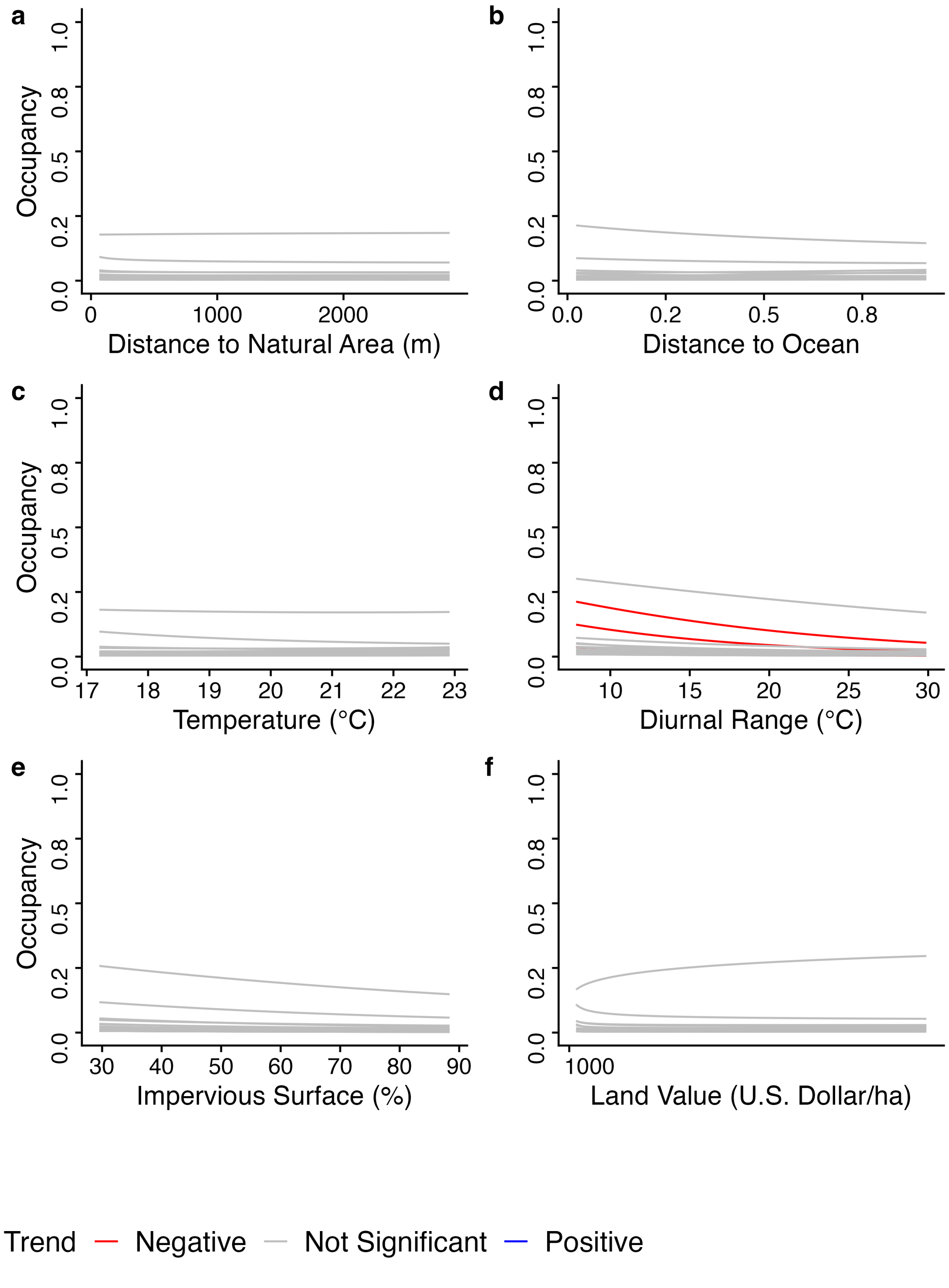

Figure S11. Within-group predictors of occupancy in Tipuloidea. 20 species are present in this group. Each plot is showing the single effect of an environmental variable by keeping the other environmental variables in the same model at their average value from the sampling data. Each line represents the effect of the environmental variable on an individual species. The color of the line represents a negative (red), not significant (grey), or positive (blue) correlation between the occupancy of a species and an environmental variable.

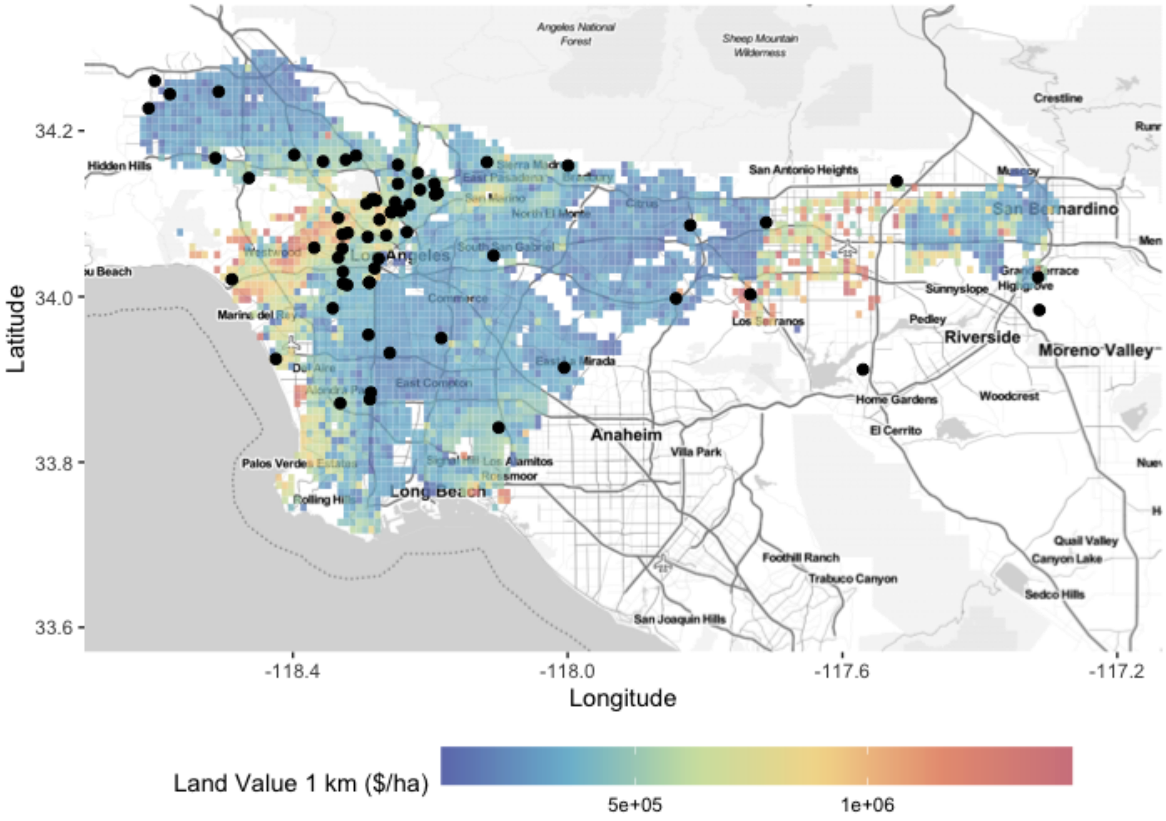

Figure S12. Map of area-weighted residential land value data, aggregated to a 1 km grid, aligned to the other spatial data layers, and masked to the extent of the study. The underlying raw data was used for neighborhood estimates of land value at 2 spatial scales: 0.25 km and 0.5 km, and used in our occupancy models. The spatial data represented here is based on the same underlying data, but aggregated to 1 km for visualization purposes for the richness interpolation shown in Figure 1. R packages ggplot2, ggmap, and basemaps were used to create this map. Map tiles by Stamen Design, under CC BY 4.0. Data by OpenStreetMap, under ODbL.

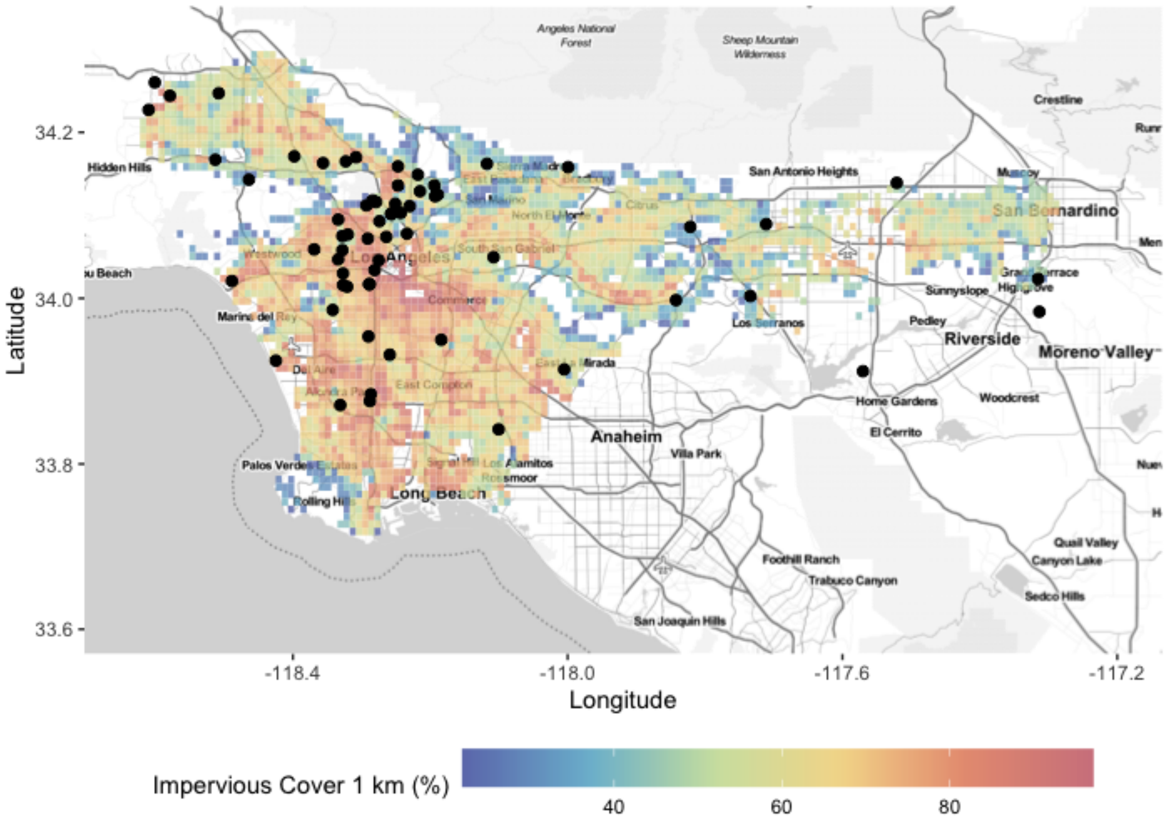

Figure S13. Map of impervious cover percentage, aggregated to a 1 km grid, aligned to the other spatial data layers, and masked to the extent of the study. The underlying raw data was used for neighborhood estimates of impervious cover at 2 spatial scales: 0.25 km and 0.5 km, and used in our occupancy models. The spatial data represented here is based on the same underlying data, but aggregated to 1 km for visualization purposes for the richness interpolation shown in Figure 1. R packages ggplot2, ggmap, and basemaps were used to create this map. Map tiles by Stamen Design, under CC BY 4.0. Data by OpenStreetMap, under ODbL.

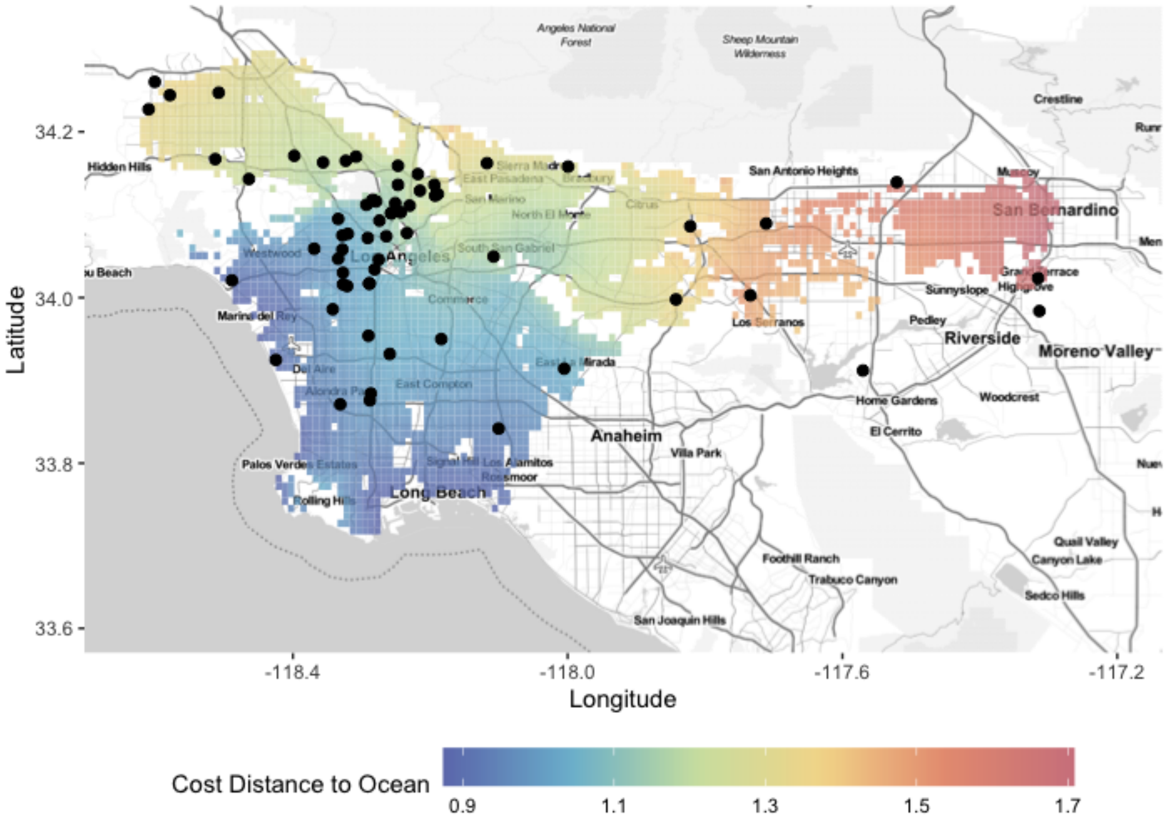

Figure S14. Map of cost distance to ocean, aggregated to a 1 km grid, aligned to the other spatial data layers, and masked to the extent of the study. The spatial data represented here is based on the same underlying data as used for site-level estimates in our occupancy models, but presented in a different format and used for the richness interpolation shown in Figure 1. See Methods for explanation of the derivation of this layer.R packages ggplot2, ggmap, and basemaps were used to create this map. Map tiles by Stamen Design, under CC BY 4.0. Data by OpenStreetMap, under ODbL.

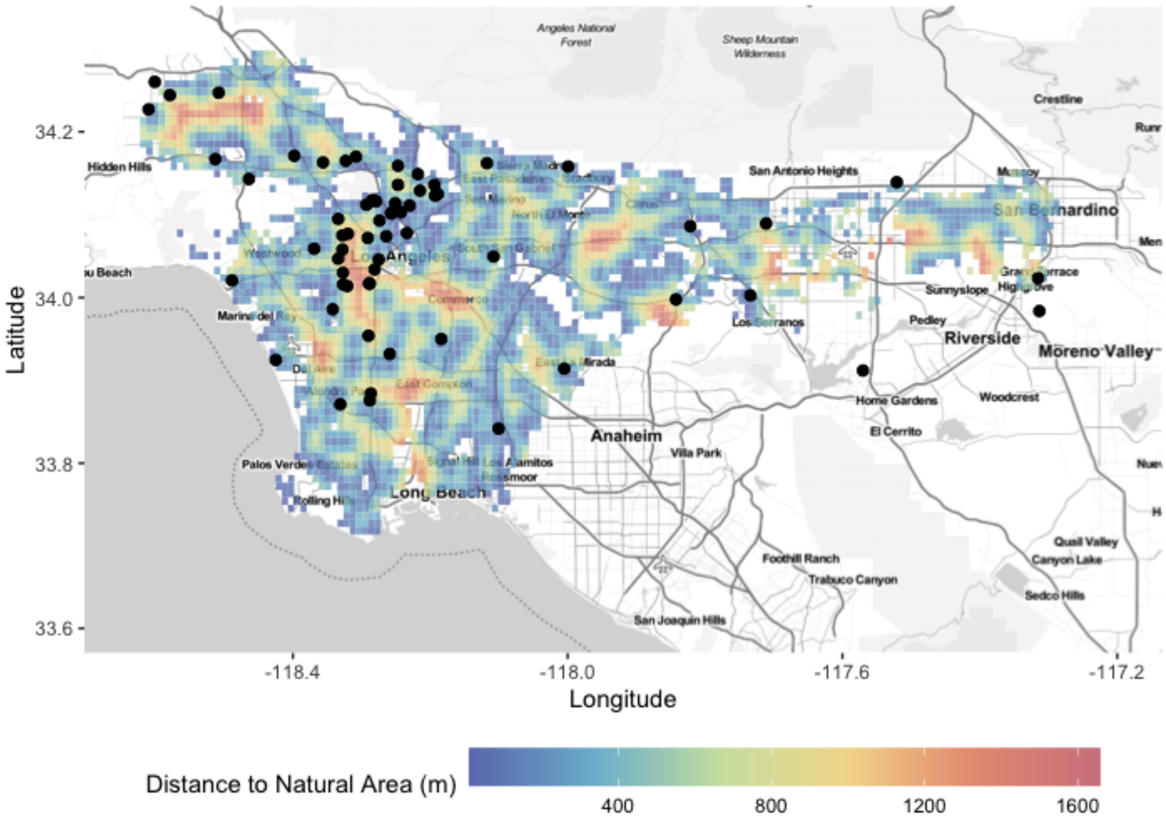

Figure S15. Map of distance to natural areas, aggregated to a 1 km grid, aligned to the other spatial data layers, and masked to the extent of the study. The spatial data represented here is based on the same underlying data as used for site-level estimates in our occupancy models, but presented in a different format and used for the richness interpolation shown in Figure 1. See Methods for explanation of the derivation of this layer. R packages ggplot2, ggmap, and basemaps were used to create this map. Map tiles by Stamen Design, under CC BY 4.0. Data by OpenStreetMap, under ODbL.

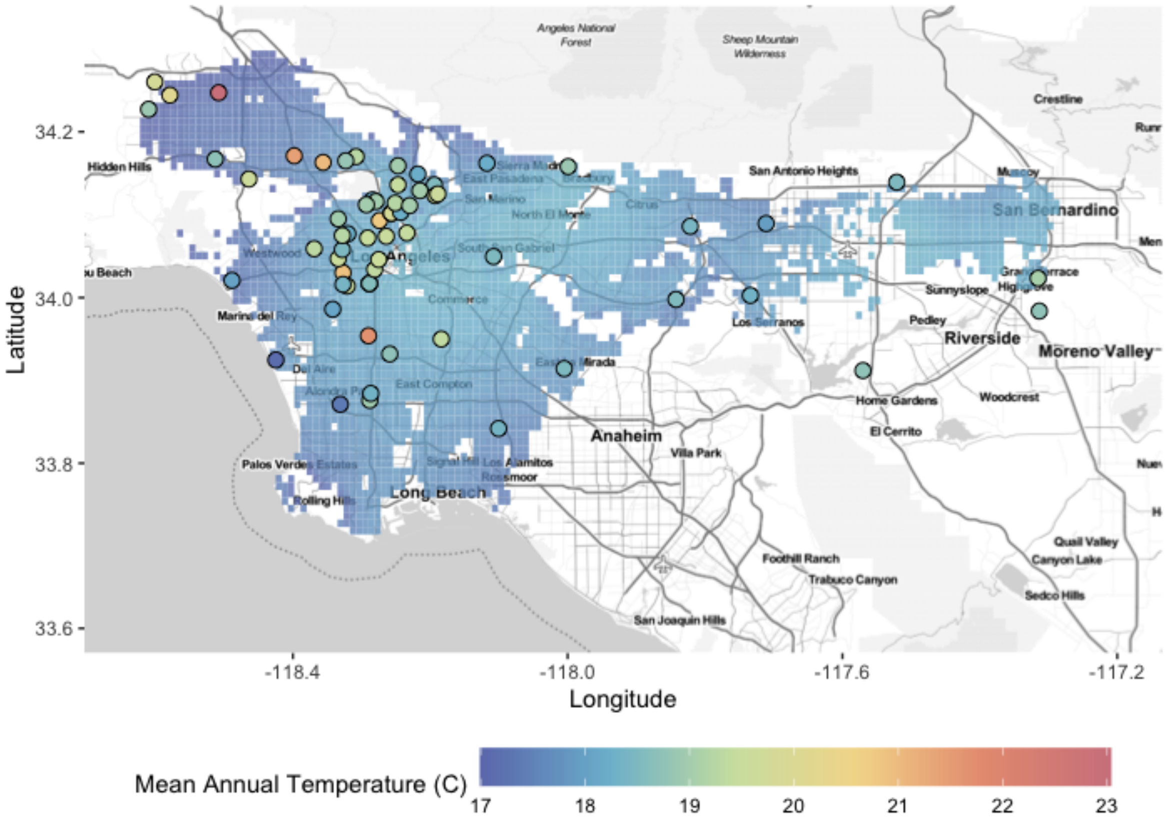

Figure S16. Map of mean annual temperature, aligned to the other spatial data layers and masked to the extent of the study. The raster data presented here is from WorldClim, while the overlaid points are the site-level values gathered from HOBO sensors present at our sampling sites and used in the occupancy models. The raster data here was used for the richness interpolation shown in Figure 1 after re-scaling. R packages ggplot2, ggmap, and basemaps were used to create this map. Map tiles by Stamen Design, under CC BY 4.0. Data by OpenStreetMap, under ODbL.

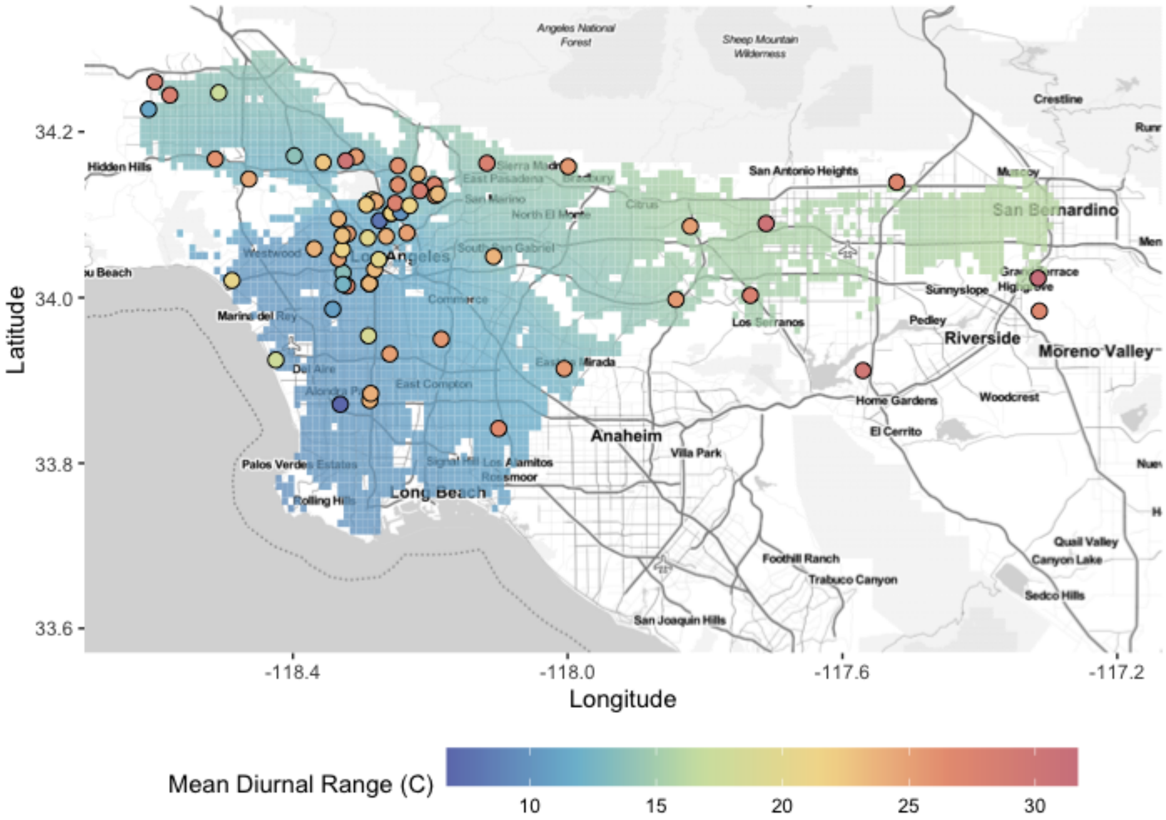

Figure S17. Map of mean diurnal temperature range, aligned to the other spatial data layers and masked to the extent of the study. The raster data presented here is from WorldClim, while the overlaid points are the site-level values gathered from HOBO sensors present at our sampling sites and used in the occupancy models. The raster data here was used for the richness interpolation shown in Figure 1 after re-scaling. R packages ggplot2, ggmap, and basemaps were used to create this map. Map tiles by Stamen Design, under CC BY 4.0. Data by OpenStreetMap, under ODbL.
